# Novel Targeting to XCR1^+^ Dendritic Cells Using Allogeneic T Cells for Polytopical Antibody Responses in the Lymph Nodes

**DOI:** 10.3389/fimmu.2019.01195

**Published:** 2019-05-29

**Authors:** Yusuke Kitazawa, Hisashi Ueta, Yasushi Sawanobori, Tomoya Katakai, Hiroyuki Yoneyama, Satoshi Ueha, Kouji Matsushima, Nobuko Tokuda, Kenjiro Matsuno

**Affiliations:** ^1^Department of Anatomy (Macro), School of Medicine, Dokkyo Medical University, Tochigi, Japan; ^2^Department of Immunology, Graduate School of Medical and Dental Sciences, Niigata University, Niigata, Japan; ^3^TME Therapeutics Inc., Tokyo, Japan; ^4^Division of Molecular Regulation of Inflammatory and Immune Diseases, Research Institute for Biomedical Sciences, Tokyo University of Science, Chiba, Japan

**Keywords:** dendritic cell targeting, allogeneic T cell, vaccination, asplenia, polytopical antibody production, lymph nodes, XCR1+ dendritic cell

## Abstract

Vaccination strategy that induce efficient antibody responses polytopically in most lymph nodes (LNs) against infections has not been established yet. Because donor-specific blood transfusion induces anti-donor class I MHC antibody production in splenectomized rats, we examined the mechanism and significance of this response. Among the donor blood components, T cells were the most efficient immunogens, inducing recipient T cell and B cell proliferative responses not only in the spleen, but also in the peripheral and gut LNs. Donor T cells soon migrated to the splenic T cell area and the LNs, with a temporary significant increase in recipient NK cells. XCR1^+^ resident dendritic cells (DCs), but not XCR1^−^ DCs, selectively phagocytosed donor class I MHC^+^ fragments after 1 day. After 1.5 days, both DC subsets formed clusters with recipient CD4^+^ T cells, which proliferated within these clusters. Inhibition of donor T cell migration or depletion of NK cells by pretreatment with pertussis toxin or anti-asialoGM_1_ antibody, respectively, significantly suppressed DC phagocytosis and subsequent immune responses. Three allogeneic strains with different NK activities had the same response but with different intensity. Donor T cell proliferation was not required, indicating that the graft vs. host reaction is dispensable. Intravenous transfer of antigen-labeled and mitotic inhibitor-treated allogeneic, but not syngeneic, T cells induced a polytopical antibody response to labeled antigens in the LNs of splenectomized rats. These results demonstrate a novel mechanism of alloresponses polytopically in the secondary lymphoid organs (SLOs) induced by allogeneic T cells. Donor T cells behave as self-migratory antigen ferries to be delivered to resident XCR1^+^ DCs with negligible commitment of migratory DCs. Allogeneic T cells may be clinically applicable as vaccine vectors for polytopical prophylactic antibody production even in asplenic or hyposplenic individuals.

## Introduction

For vaccination against infectious agents, efficient antigen delivery to sites of T cell priming within the recipient secondary lymphoid organs (SLOs) is crucial for the successful production of neutralizing antibodies (1). As dendritic cells (DCs) are professional antigen presenting cells, the targeting of antigens to DCs has been investigated extensively as therapeutic vaccines (2, 3).

Major sites of immune response after vaccination are generally the spleen and regional LNs. Because the spleen is crucial for antibody production against antigens in the blood, patients with an absent or dysfunctional spleen are at increased risk of severe infection. Intense vaccination against encapsulated bacteria and pandemic viruses as well as prophylactic antibiotics therapy are strongly recommended for splenectomized patients (4), and the development of a vaccination method for these patients that induces an efficient antibody response in other SLOs is a clinically important issue. Since a normal young adult body contains up to 450 LNs (5), the antigen targeting to DCs polytopically in most LNs would be able to promote intense antibody production.

However, systemic induction of an antibody response in LNs throughout the body is very difficult. Antigens enter the regional LNs generally via the draining lymphatics from the peripheral organs and, consequently, the immune response is only induced locally (6). The exception is live vaccines, which act similar to natural infection, rapidly replicating and disseminating throughout the vascular network and launching multiple immune response foci in the SLOs that drain the target organs (1). In contrast, entry of soluble antigens from the blood via the high endothelial venules (HEVs) hardly occurs (7). There has been one report of an antigen and OTII cell response in the peripheral and mesenteric LNs following intravenous (i.v.) administration of soluble FITC-labeled OVA into mice that received OTII cells in advance (8). However, the molecular weight of OVA is low, 43 kDa, and this protein antigen may have entered the afferent lymph from the periphery and accumulated in the regional LNs in the same fashion as other plasma proteins (9). In addition, the immunogenicity of OVA is weak and only detectable by enhanced detection methods using OTI or OTII cells, and additional strong adjuvant would be necessary (10).

In a steady state, only DC precursors seeding the SLOs (11) and mouse plasmacytoid DCs (12, 13) can home to the LNs. Previously, we demonstrate the presence of a unique immature DC population in the liver (14, 15) and bone marrow (14) that can transmigrate through the blood vessel wall to the SLOs. We define these cells as immature DCs that can perform blood-borne migration through the blood vessel wall of the SLOs. In contrast, when mature splenic DCs (16, 17) or allogeneic lymph DCs (18–20) are transferred i.v., these cells are almost completely excluded from SLOs other than the spleen. DCs in the peripheral organs can only enter the regional LNs via the afferent lymphatics (21). Thus, designing DC-targeted non-live vaccines or antigen-pulsed DCs that are able to induce polytopical antibody production in the SLOs by i.v. administration has been almost impossible, and to the best of our knowledge no report is yet available.

Donor-specific transfusion (DST), which simply transfuses fresh donor blood i.v. prior to transplantation, is one of the tolerance-inducing protocols used in not only experimental (22), but also clinical (23) transplantation. Recently, we demonstrated that a single DST efficiently induces a helper T cell-dependent anti-donor class I MHC (MHCI) antibody-forming cell and donor-specific regulatory T cell responses, mainly in the spleen (24). Notably, we also found that these antibodies were still produced after DST even in splenectomized recipients. Thus, SLOs other than the spleen can respond to i.v. injection of donor blood. The findings suggest the presence of alloantigen-transporting cells in the blood that could migrate to the SLOs after DST. Therefore, it is of interest to examine which components of the donor blood, i.e., white blood cells (WBCs), platelets, red blood cells (RBCs), or blood plasma, can induce the DST response and how the response is induced in the recipient SLOs.

In the present study, we examined the mechanism underlying this efficient DST response in rats. We demonstrate that donor blood T cells are the most proficient immunogens, and their entrance into the T cell areas and DST responses to donor MHCI occur polytopically not only in the spleen, but also in the LNs. A clinical implication is discussed concerning antigen-furnished T-cells as i.v. applicable vaccine vector for the prophylactic antibody production polytopically in the LNs even in the absence of the spleen.

## Results

### Donor T Cells Are the Most Effective Inducers of the DST Antibody Response

**Figure 1:**
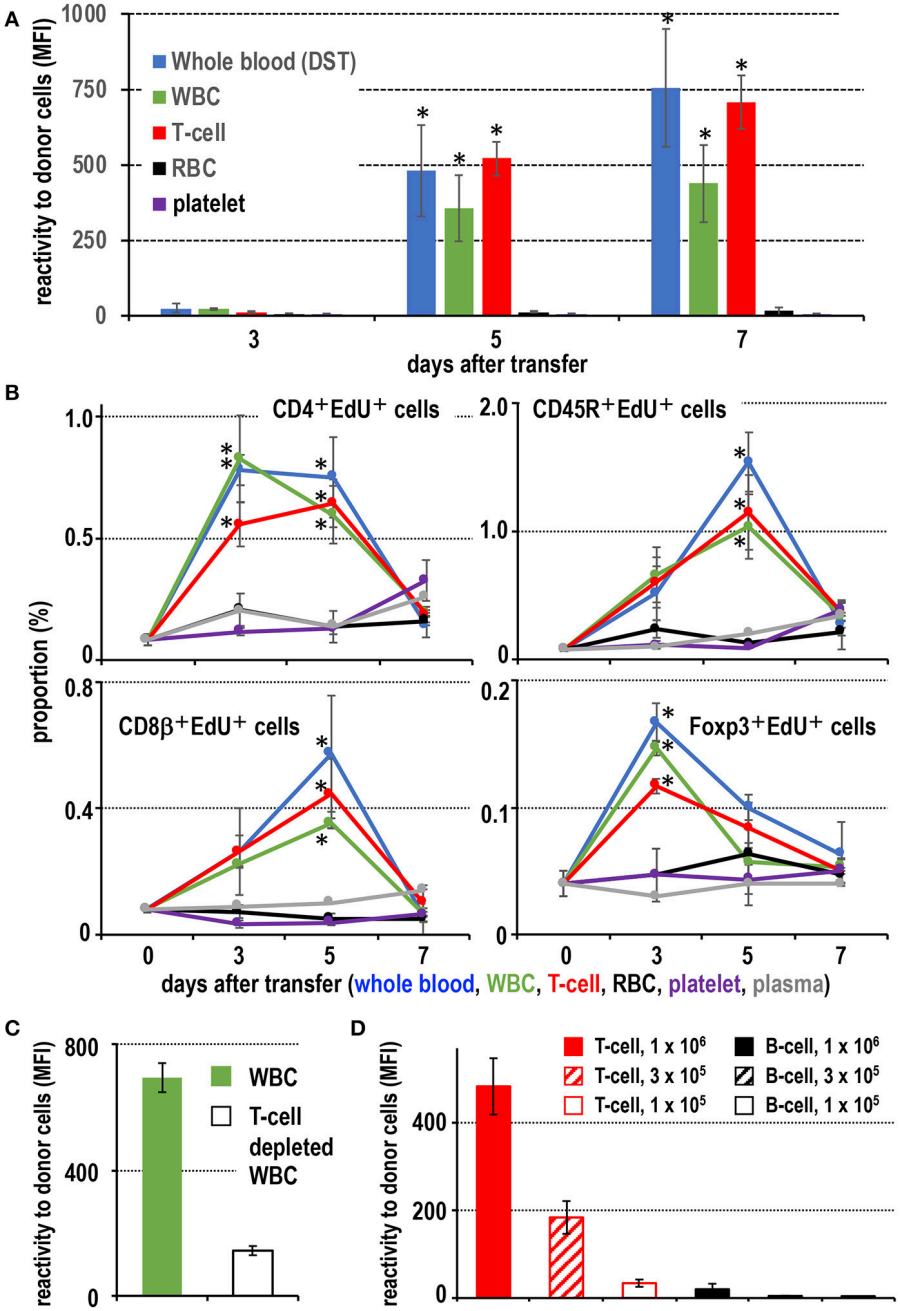
Screening of effective blood components for the donor-specific transfusion (DST) response. **(A)** Production of DST antibodies in recipient blood after donor blood component injection. In the white blood cell (WBC) and T cell injected groups, DST antibodies were detected after 5 days (mean ± SD, *n* = 3 rats each, *P < 0.05). **(B)** The time kinetic changes in each proliferating cell type as a proportion of total splenocytes. Two-color FCM analysis of recipient splenic lymphocytes after donor blood component injection for proliferating cells (EdU^+^) and CD4^+^ T cells, CD8β^+^ T cells, CD45R^+^ B cells, or regulatory T cells (Foxp3^+^). The proliferation of CD4^+^ T cells and regulatory T cells peaked 3 days after treatment, whereas B cells and CD8β^+^ T cells peaked at 5 days. These results were similar to the DST group that received whole blood (mean ± SD, *n* = 3 rats each, *P < 0.05). **(C)** With T cell depletion of WBCs, the DST antibody production was suppressed at 7 days. **(D)** Dose-dependent response following injection of isolated T cells or B cells. T cells induced much higher DST antibody production than B cells (mean ± SD, *n* = 3 rats each, *P < 0.05). MFI, mean fluorescent intensity.

When fresh blood of ACI rats was transfused to Lewis rats, an equivalent number of purified T cells and WBC fractions present in the blood induced a comparable level of DST antibodies (Figure 1A). Flow cytometric analysis (FCM) of recipient spleen cells revealed similar proliferative responses of CD4^+^ T cells, Foxp3^+^ regulatory T cells, and CD45R^+^ B cells as in those who received whole blood (Figure 1B). In contrast, transfusion of other components, such as purified RBCs, platelets, or blood plasma, induced a significantly delayed and less intense response (Figures 1A,B). Furthermore, when T cells were depleted from the WBC fractions, the serum DST antibody response was suppressed considerably at 7 days (Figure 1C).

For dose response, we employed thoracic duct lymphocytes (TDLs) as a convenient source of donor lymphocytes, which were functionally almost the same as those in the blood (25, 26). At least 3 × 10^5^ T cells from donor TDLs, representing those in ~100 μl of blood, induced significant DST antibody production (Figure 1D). In contrast, 10^6^ B cells induced a significant but very low level comparable to that induced by 1 × 10^5^ T cells (Figure 1D). When equivalent numbers of purified CD4^+^ T cells and CD8^+^ T cells ware transferred, both induced comparable DST antibody production (Figure S1), indicating both subsets are equally efficient.

These results indicate that T cells are the most proficient immunogens for the DST response among blood components. A pure T cell fraction, both CD4^+^ and CD8^+^, without DCs or monocytes (equivalent to ~100 μl blood) is enough for significant induction of a DST antibody response in the ACI to Lewis rat combination, and B cells require ~10-times as many cells.

### Fate of Donor Cells in the Spleen and the LNs

**Figure 2:**
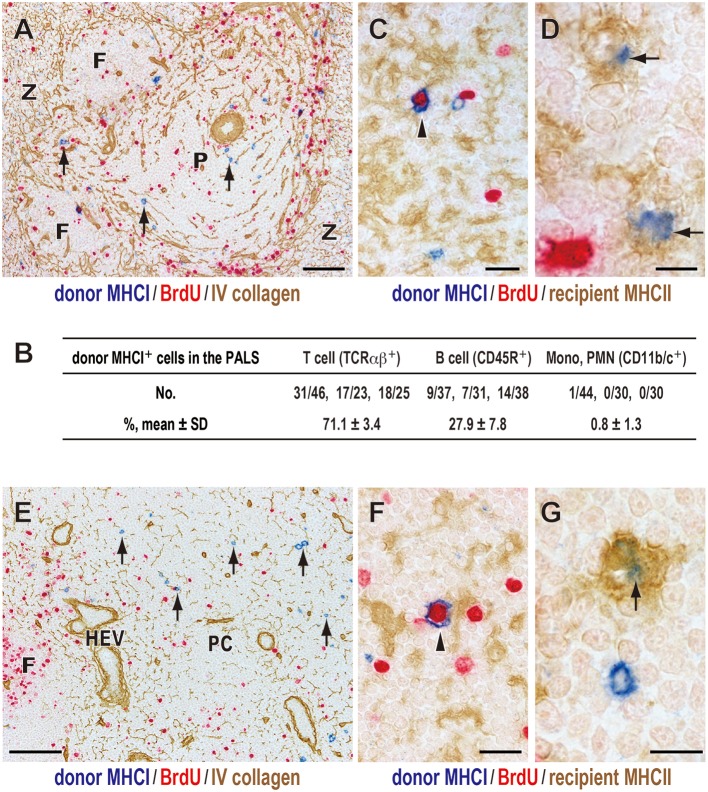
Fate of donor T cells after DST. Three-color immunostaining of recipient spleens **(A–D)** or peripheral LNs **(E–G)** for donor MHCI (blue), BrdU (red), and type IV collagen (brown) or recipient MHCII (brown). **(A,E)** Donor MHCI^+^ cells (arrows) were observed in the PALS **(A)** and T cell area of the LNs (paracortex, PC) **(E)** at day 1. **(B)** Phenotype of donor MHCI^+^ cells and cell ratio in the PALS. Approximately 100 donor MHCI^+^ cells/rat were examined (*n* = 3). Donor MHCI^+^ cells that migrated to the PALS were TCRαβ^+^ T cells (~70%) and CD45R^+^ B cells (~30%). **(C,F)** A donor cell binding to MHCII^+^ putative recipient DCs (brown) became BrdU^+^ (arrowhead) in the PALS **(C)** and the PC **(F)** at day 1, representing a graft vs. host reaction. **(D,G)** Donor MHCI^+^ fragments (blue) superimposed on the cytoplasm of recipient putative DCs (arrows) in the PALS **(D)** and the PC **(G)** at day 2, suggesting phagocytosis. F, lymph follicle; P, splenic PALS; Z, marginal zone; HEV, high endothelial venule. Scale bar = 100 μm **(A,E)**, 20 μm **(C,F)**, or 10 μm **(D,G)**.

Immunohistological examination of recipient spleens found that, 1 day after DST, donor MHCI^+^ cells were found in the splenic T cell area, the periarterial lymphocyte sheath (PALS) (Figure 2A), which consisted mainly T cells (71.1 ± 3.4%) and B cells (27.9 ± 7.8%) but few other cell types (Figure 2B). This migration was very fast and readily observed by 15 min after DST, as reported previously (27).

Many of migrated T cells bound to recipient class II MHC (MHCII)^+^ cells with dendritic cytology in the PALS, and some became large in size and BrdU^+^ (Figure 2C). As these MHCII^+^ cells are mostly CD103^+^CD11b^+^ resident interdigitating DCs (14), the result indicates a graft vs. host (GvH) reaction via the direct pathway in which donor T cells become activated within the cluster with recipient DCs (14, 27).

Donor MHCI^+^ cells decreased in number at 2 days and almost disappeared by 3 days. From day 1, donor MHCI^+^ fragments appeared in the PALS, and at day 2 they often superimposed on the cytoplasm of some recipient MHCII^+^ cells with dendritic cytology (Figure 2D). This suggests phagocytosis of donor MHCI^+^ fragments by recipient DCs in the PALS.

As for the LNs, donor MHCI^+^ cells also readily migrated to the T cell area of LNs, paracortex, and showed almost the same kinetics as that in the spleen (Figures 2E–G).

### Donor T Cells Fragments Are Phagocytosed by XCR1^+^ Resident DCs in the PALS

To confirm phagocytosis of donor MHCI fragments and the phenotype of phagocytic DCs, we examined subsets of conventional DCs that were CD103^+^MHCII^+^ in the normal rat spleen (28, 29). FCM detected two subsets, CD4^−^XCR1^+^signal regulatory protein 1 α (SIRP1α)^−^CD200^+^ cells and CD4^+^XCR1^−^SIRP1α^+^CD200^−^ cells (Figure S2A). SIRP1α is CD172a. Because mouse CD8^+^ DCs were recently defined as XCR1^+^SIRP1α^−^ (30, 31), it is reasonable to consider the CD4^−^XCR1^+^SIRP1α^−^CD200^+^ subset as the rat counterpart of mouse CD8^+^ DCs. These subsets were detected by immunohistology in the PALS and a CD103^+^XCR1^+^ subset constituted 40.1 ± 6.9% in a normal steady state (Figures S2C,E).

In mice, XCR1 are exclusively expressed by CD8^+^DC subset (31). In rats, however, the mAb to XCR1 reacted with not only DCs but also CD103^−^ non-DCs (Figure S2C). Double immunostaining of rat spleens for XCR1 and a macrophage marker CD169 (Figure S2D) or CD163 (not shown) revealed a presence of XCR1^+^CD169^+^ cells and XCR1^+^CD163^+^ cells in the marginal zone and red pulp, respectively. CD169^+^ cells in the outer margin of the PALS and in the marginal zone are marginal metallophilic macrophages and marginal zone macrophages, respectively and CD163^+^ cells are red pulp macrophages (27, 32). The mAb we used exclusively reacted with mice DCs (not shown), while isotype control of the XCR1 mAb (IgG_2b_) did not detect CD103^+^ DCs or CD169^+^ and CD163^+^ macrophages (Figure S2D), confirming the specificity of the mAb. Accordingly, the results indicate that at least some macrophages in the rat spleen also express XCR1.

**Figure 3:**
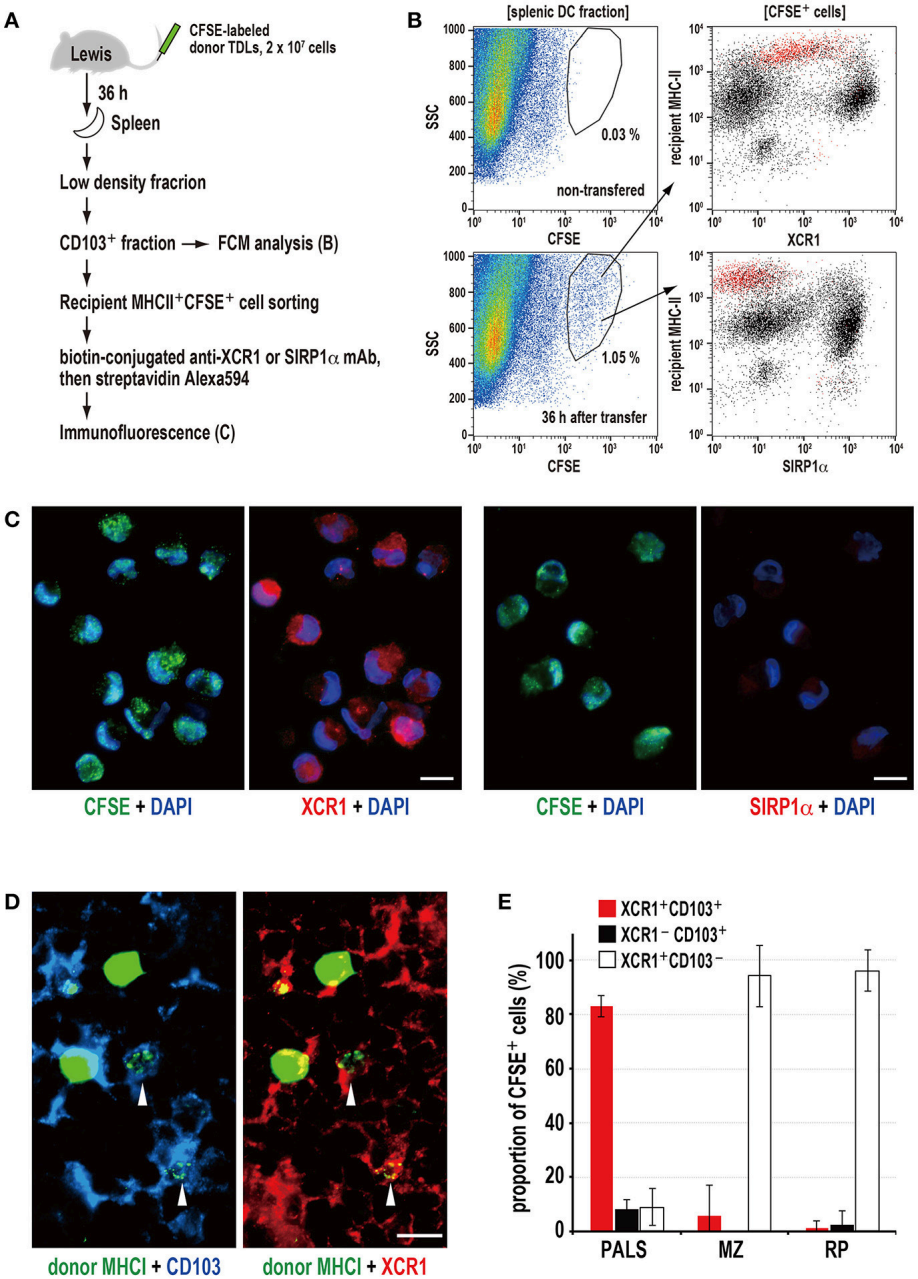
Phagocytosis of donor MHCI fragments and phenotype of phagocytic DCs in the spleen. **(A)** Experimental protocol for identifying DCs that phagocytosed donor cells. **(B)** Three-color FCM analysis of magnetically isolated CD103^+^ DC fraction of the recipient spleen 36 h after CFSE-labeled thoracic duct lymphocyte (TDL) injection for CFSE, XCR1, or SIRP1α, and recipient MHCII. XCR1^+^SIRP1α^−^CD103^+^MHCII^high^ DCs expressing CFSE^+^ signals (red dots) were detected within the gated fraction (1.05%) of the SSC vs. CFSE plot. A representative data of 4 individual experiments. **(C)** Two-color immunofluorescent staining of cytosmears of sorted CD103^+^recipient MHCII^high^CFSE^+^ cells for DAPI (DNA stain, blue), and XCRI or SIRP1α (red). CFSE^+^ donor cell fragments (green) were observed in the cytoplasm of XCR1^+^SIRP1α^−^ DCs but not XCR1^−^SIRP1α^+^ DCs, indicating phagocytosis. Scale bar = 10 μm. **(D)** Three-color immunofluorescent staining of the PALS cryosections for donor MHCI (green), CD103 (blue), and XCR1 (red). The arrowheads indicate XCR1^+^CD103^+^ DCs with phagocytosed donor MHCI^+^ fragments. Scale bar = 10 μm. **(E)** The subset and ratio of CFSE^+^ phagocytic cells in different splenic areas. In the PALS, XCR1^+^CD103^+^ DCs accounted for more than 80% in CFSE^+^ phagocytic cells (mean ± SD, *n* = 4 rats each). In contrast, more than 90% phagocytic cells in the marginal zone (MZ) and red pulp (RP) were XCR1^+^CD103^−^ non-DCs.

Next, we examined isolated spleen cells and cryosections 36 h after transfer of CFSE-labeled donor TDLs (Figure 3A). FCM revealed that CD103^+^ fractions contained CFSE^+^ signals, and that these cells were CD103^+^MHCII^high^XCR1^+^SIRP1α^−^ DCs (Figure 3B). When the CFSE^+^CD103^+^MHCII^high^ DC fraction was sorted and cytosmeared, these DCs were XCR1^+^SIRP1α^−^ and contained cytoplasmic donor CFSE^+^ fragments (Figure 3C), demonstrating phagocytosis of donor cell fragments.

Of note, the XCR1^+^ DCs could be separated into two populations according to their phagocytic activities and phenotype, where phagocytic cells were mostly XCR1^low~int^MHCII^high^CD103^+^ and non-phagocytic cells were mostly XCR1^high^MHCII^int^CD103^+^, respectively (Figure 3B). We do not know whether this difference is due to phagocytic events or not, because untreated rat spleen also contained these two populations (Figure S2B).

Immunohistology revealed donor MHCI^+^ fragments superimposed on CD103^+^XCR1^+^ DCs in the PALS (Figure 3D), representing phagocytosis. When the phenotypes of phagocytic cells in the different components of the spleen were examined, more than 80% of the cells in the PALS were CD103^+^XCR1^+^ DCs (Figure 3E). Although CD103^+^XCR1^+^ or CD103^+^XCR1^−^ DCs were also present in the marginal zone and the red pulp, phagocytosis of the donor MHCI^+^ fragments was negligible (Figure 3E).

These data demonstrate that XCR1^+^SIRP1α^−^MHCII^high^CD103^+^ resident DCs selectively phagocytose donor cell fragments in the PALS and that XCR1^+^ macrophages outside of the PALS also phagocytose them as scavengers.

### NK Cells Are Responsible for Donor Cell Phagocytosis in the PALS

**Figure 4:**
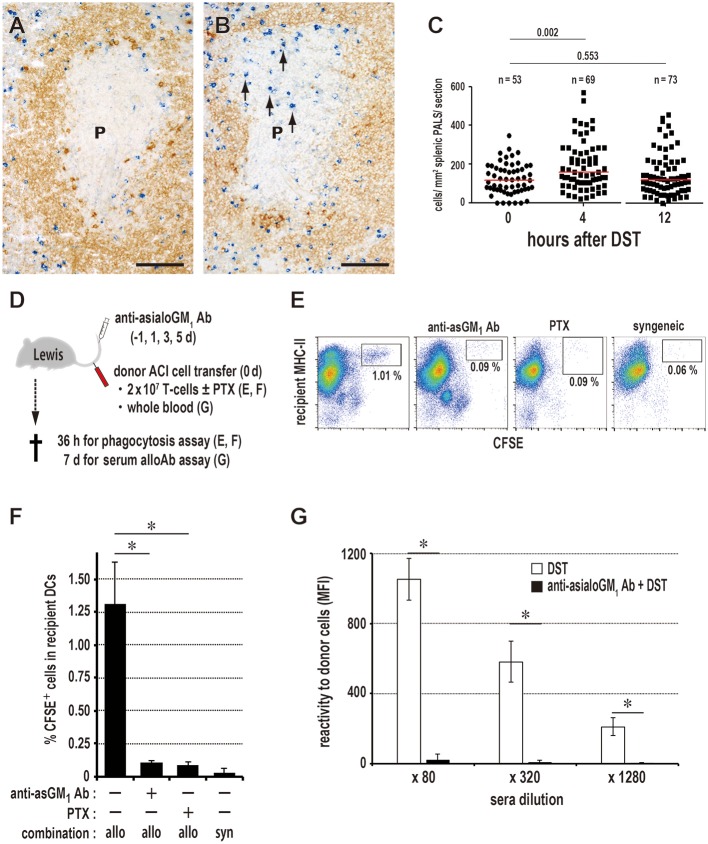
Kinetics of NK cells and donor cell phagocytosis in the PALS. Double immunostaining for asialo GM_1_ (blue) and rat IgM (brown) in the splenic PALS 0 **(A)** and 4 h **(B)** after donor-specific transfusion (DST). Arrows indicate asialo GM${}_{1}^{+}$ NK cells. (P, splenic PALS. Scale bar = 100 μm. **(C)** Number of asialo GM${}_{1}^{+}$ NK cells/mm^2^ of PALS 0, 4, and 12 h after DST. NK cells significantly increased in the PALS 4 h after DST. Data was analyzed by Mann-Whitney *U*-test. **(D)** Experimental protocol for examining the effect of NK cell depletion by anti-asialo GM_1_ antibody (Ab) on the DST response. PTX, pertussis toxin. **(E,F)** With NK cell depletion, DCs ingesting CFSE^+^ donor T cell fragments in the spleen significantly decreased in number to the same level of the syngeneic combination group. PTX pretreatment of donor T cells also resulted in the significant decrease. mean ± SD, *n* = 3 rats each, *P < 0.05. allo, allogeneic ACI to Lewis; syn, syngeneic Lewis to Lewis. **(G)** In the NK cell-depleted group, the DST antibody response disappeared completely (mean ± SD, *n* = 3 rats each, *P < 0.05). MFI, mean fluorescent intensity.

The number of asialo GM${}_{1}^{+}$ NK cells/mm^2^ of PALS significantly increased 4 h after DST (Figures 4A–C). After NK cell depletion with anti-asialo GM_1_ antibody, CD103^+^XCR1^+^ DCs ingesting CFSE^+^ donor T cell fragments significantly decreased in number (Figures 4D–F). The production of serum DST antibodies 7 days after DST was suppressed almost completely (Figure 4G). Although we performed quantitative PCR (qPCR) analysis of the whole spleen shortly after the donor T cell transfer, we could not detect increased mRNA levels of NK-recruiting chemokines (Figure S3A), probably because the increase in NK cell number was small, ~1.5-fold.

Concerning NK activity against allogeneic cells (33), other two rat strains, PvG/c and BN rats, which readily showed the DST antibody response by 7 d after DST treatment, were compared as recipients (Figures S4A,B). ACI rats were used as donors. PvG/c rats clearly exhibited accelerated phagocytosis by XCR1^+^ DCs, as it occurred 1 day earlier than with Lewis rats (Figures S4C,G). When BN rats were used as recipients, donor T cells (Figure S4D) extensively proliferated (Figure S4E), indicating a predominance of GvH reaction, probably due to low NK activity. Because this causes dilution of the CFSE^+^ signals of donor T cells, detection of phagocytosis becomes difficult. Accordingly, we transferred mitomycin C (MMC)-treated donor T cells to inhibit their proliferation. With this, donor T cells disappeared (Figure S4F) and we readily observed phagocytosis at 1.5 days (Figure S4H). These results indicate that NK activities correlated well with the phagocytosis by XCR1^+^ DCs in at least three rat strain combinations (Figure S4I). As strain differences in NK reactivity is a rather complex issue, other mechanisms of donor cell killing may be involved in other rat strain combinations.

Alternatively, CD161a^+^ (NK1.1^+^) killer DCs have been reported in the rat spleen (28) and effector/memory fraction of CD8^+^ T-cells is known to be asialo GM${}_{1}^{+}$ in mice (34). These cells may be related to donor cell phagocytosis. Although two subsets of splenic DCs were both CD11b^+^CD161a^+^, they were asialo GM${}_{1}^{-}$ (Figure S3C) and not depleted by anti-asialo GM_1_ antibody (data not shown). Also, we found that in rats, CD8^+^ T-cells and TCRαβ^+^CD161a^+^ putative NKT cells were mostly asialo GM${}_{1}^{-}$ (Figure S3D). These findings suggest that rat NK cells are mostly asialo GM${}_{1}^{+}$CD161a^+^. Because asialo GM${}_{1}^{+}$ cells are either CD8α^+^ or CD8α^−^ (Figure S3D), NK cells can be further divided into two subsets. In fact, anti-CD8α monoclonal antibody (mAb) treatment resulted in only partial depletion of NK cells (35) and partial suppression of phagocytosis (unpublished data, Ueta). Therefore, the results indicate that involvement of killer DCs or CD8^+^ T-cells would be minor for the donor cell phagocytosis.

### Migration of Donor T Cells to the PALS Is Crucial for the DST Response

**Figure 5:**
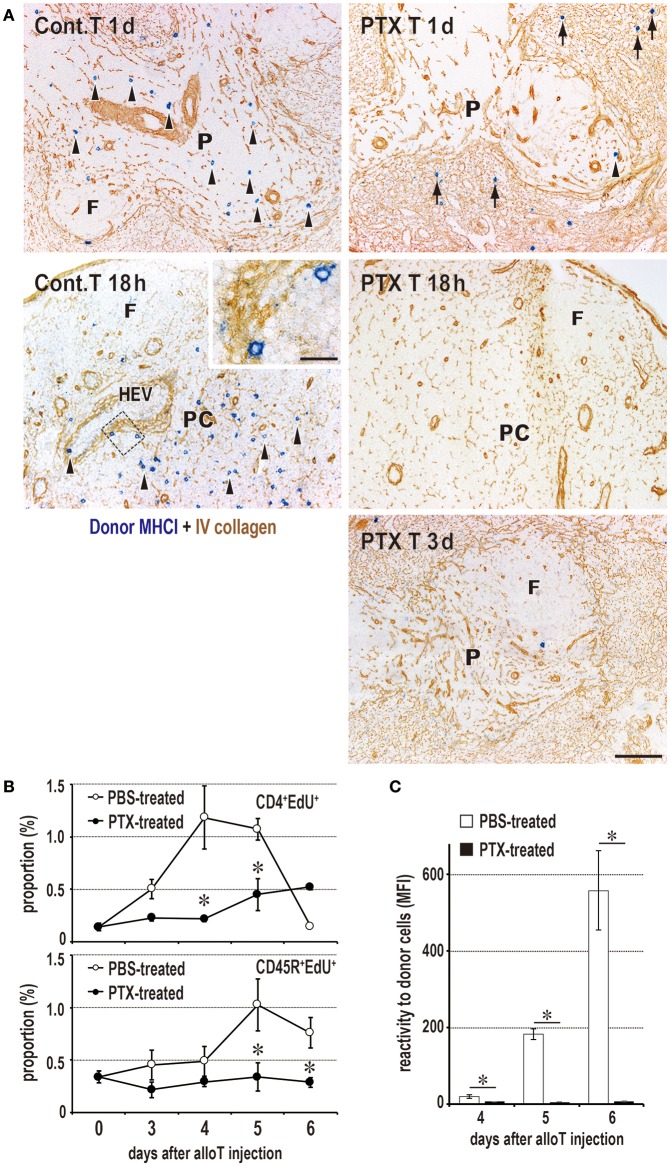
Requirement of T cell migration to the PALS for induction of the DST response. **(A)** Two-color immunostaining for donor MHCI (blue) and type IV collagen (brown) in the spleen at 1 and 3 days after 2 × 10^6^ donor T cell injection and in the peripheral LNs at 18 h after 2 × 10^7^ donor T cell injection. The control group exhibited migration to the PALS by 1 day (Cont T 1d, arrowheads) and LNs by 18 h (Cont T 18 h, arrowheads). In contrast, pertussis toxin (PTX) pretreated donor T cells did not enter the PALS up to 3 days (PTX T 3d), but stayed in the marginal zone and red pulp for 1 day (PTX T 1d, arrows). They did not enter the LNs (PTX T 18 h) either. F, lymph follicle; HEV, high endothelial venule; P, PALS; PC, paracortex. Scale bar = 100 μm or 20 μm (inset). **(B,C)** With PTX pretreatment of donor T cells, the DST responses of CD4^+^ T cells and CD45R^+^ B cells **(B)** and serum antibodies **(C)** were abrogated (mean ± SD, *n* = 3 rats each, *P < 0.05). MFI, mean fluorescent intensity.

To inhibit donor T cell migration to the PALS, we blocked the chemokine-chemokine receptor axis with pertussis toxin (PTX). Compared to the readable migration of untreated cells into the PALS, PTX-treated T cells did not enter the PALS for up to 3 days, staying in the marginal zone and red pulp for 1 day and soon disappearing (Figure 5A). In this group, DCs ingesting CFSE^+^ donor cell fragments in the spleen significantly decreased in number (Figures 4E,F), and the DST responses of CD4^+^ T cells and CD45R^+^ B cells (Figure 5B) and serum antibodies (Figure 5C) were abrogated. These results indicate that the migration of donor T cells into the PALS is indispensable for induction of the DST response.

### Donor T Cells Also Induced the DST Response in the LNs

**Figure 6:**
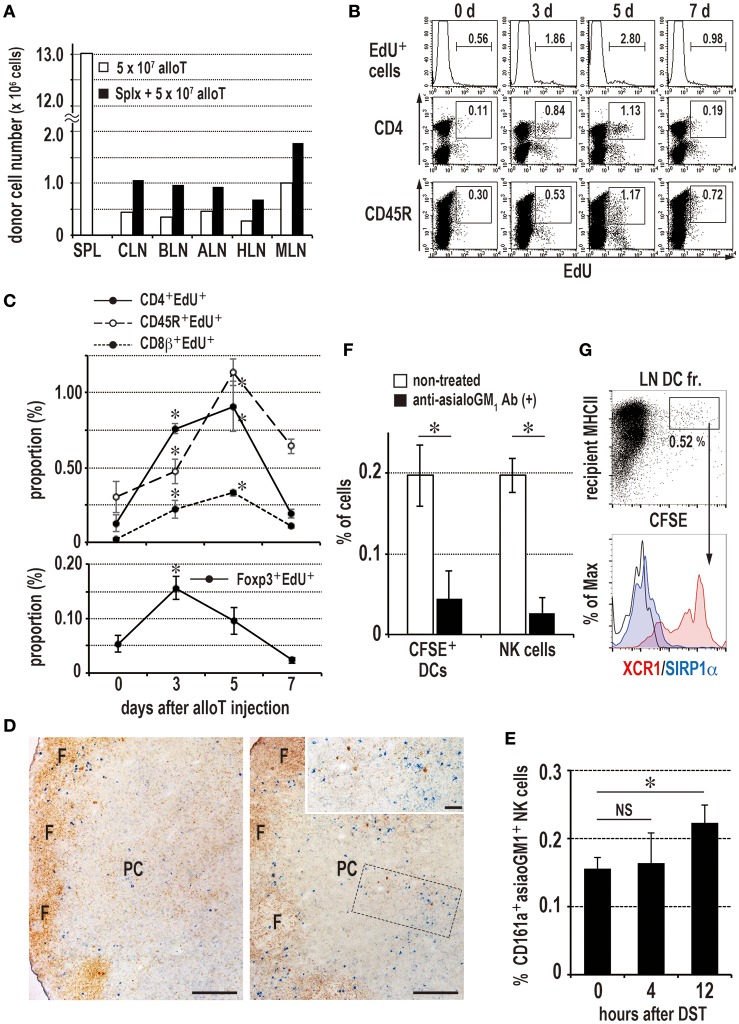
Induction of the donor-specific transfusion (DST) response in the LNs of splenectomized **(A–C,F,G)** or eusplenic **(D,E)** recipients. The recipient peripheral LNs were analyzed in the same manner as the spleen (Figure 1, 3, 4). **(A)** Two-color FCM analysis of the number of migrated donor T cells in recipient LNs 24 h after injection. Roughly twice as many donor T cells migrated to the LNs than in the eusplenic control. SPL, spleen; CLN, cervical LN; BLN, brachial LN; ALN, axillary LN; HLN, hepatic LN; MLN, mesenteric LN. **(B,C)** The time kinetic changes in each proliferating cell type/total LN cells by FCM analysis. The CD4^+^ T cells and regulatory T cells peaked at 3–5 days and 3 days, respectively, whereas CD45R^+^ B cells and CD8β^+^ T cells peaked at 5 days (mean ± SD, *n* = 3 rats each, *P < 0.05). **(D,E)** Kinetics of NK cells in the peripheral LNs after DST. **(D)** Double immunostaining of the LNs for asialo GM_1_ (blue) and rat IgM (brown) at 0 (left panel) and 12 h (right panel). Inset indicates increased asialo GM${}_{1}^{+}$ NK cells (blue) in the paracortex (PC). Scale bar = 200 μm or 50 μm (inset). F, lymph follicle. **(E)** The proportion of NK cells significantly increased in the LNs at 12 h (mean ± SD, *n* = 3 rats each, *P < 0.05). **(F)** Kinetics of donor cell phagocytosis in the peripheral LNs after donor T cell injection. Phagocytosis of donor cell fragments by DCs was detected 36 h after transfer of 2 × 10^7^ CFSE labeled T cells. With anti-asialo GM_1_ antibody (Ab) treatment, the numbers of recipient CFSE^+^ phagocytic DCs and NK cells were significantly reduced compared to non-treated control (mean ± SD, *n* = 3 rats each, *P < 0.05). **(G)** Phenotype of CFSE^+^ phagocytic DCs in the peripheral LNs. Note that they were XCR1^+^SIRP1α^−^, similar as those of spleen.

To avoid the influence of the spleen on DST responses in the LNs, rats were splenectomized just before donor T cell transfer. Donor T cells migrated to all of the examined LNs, and their number was approximately double that of eusplenic recipients (Figure 6A). The peripheral LNs (Figures 6B,C) and gut LNs (data not shown) demonstrated proliferative responses of recipient T and B cells with similar kinetics as those observed in the spleen (Figure 1B). NK cells significantly increased by 12 h (Figures 6D,E), and phagocytosis of donor cell fragments by XCR1^+^ DCs was detected in LN cell suspensions (Figures 6F,G). PTX pretreatment also suppressed donor T cell migration to the LNs (Figure 5A) and inhibited the DST antibody response (data not shown). Anti-asialo GM_1_ antibody pretreatment significantly suppressed the NK cell number and phagocytosis by XCR1^+^ DCs (Figure 6F). However, we did not detect increased mRNA levels of NK-recruiting chemokines in LN extracts (Figure S3B).

These results indicate that allogeneic T cells induced a DST antibody response in the peripheral LNs in a similar manner as in the spleen, dependent on the NK response and phagocytosis by XCR1^+^ DCs in ACI to Lewis rat combination.

### GvH Reaction Is Not Required for the DST Response

Because donor T cells exhibited a GvH reaction at 1–2 days (Figures 2C,F), we examined whether donor T cell activation is required for the DST response. T cells from (Lewis × DA)F_1_ hybrid rats (RT1.A^al^B^al^) were transferred to parental Lewis rats (RT1.A^l^B^l^), a semiallogeneic combination in which the GvH reaction does not occur. The donor T cells induced significant serum DST (anti-RT1.A^a^) antibody production on day 7 (Figure S5). Furthermore, fully allogeneic donor T cells pretreated with MMC, migrated to the SLOs and induced DST antibody production as will be described below. Thus, the GvH reaction leading to donor T cell activation and proliferation is dispensable for the DST response.

### Both XCR1^+^ and XCR1^−^ DCs Form a Cluster With Proliferating Recipient T Cells

**Figure 7:**
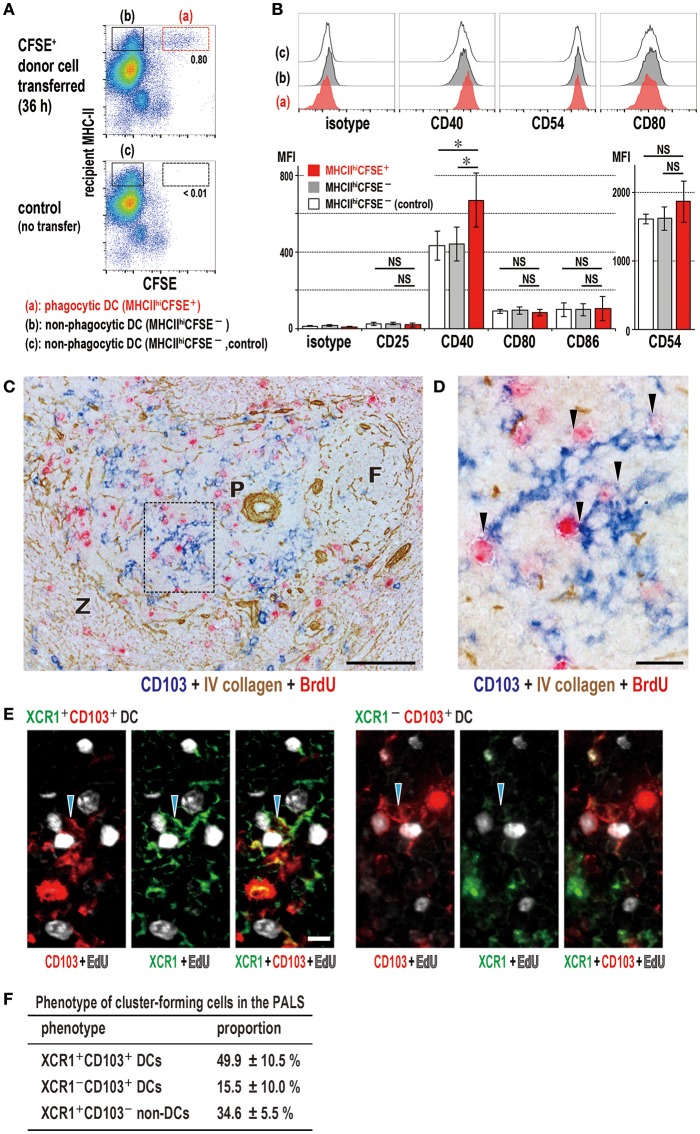
Activation state of phagocytic dendritic cells (DCs) and phenotype of DCs that cluster with proliferating cells in the PALS. **(A,B)** Upregulation of CD40 in XCR1^+^ DCs following phagocytosis. Phagocytic DCs were compared with non-phagocytic DCs in MHCII^high^ gates with/without donor cell transfer (mean ± SD, *n* = 4 rats each, *P < 0.05, NS, not significant). **(C,D)** Three-color immunostaining of the spleen 2 days after DST for CD103 (blue), BrdU (red), and type IV collagen (brown). **(C)** Many CD103^+^ DCs (blue) and BrdU^+^ proliferating cells (red) are present in the PALS (P). Scale bar = 100 μm. F, lymph follicle; Z, marginal zone. **(D)** Inset of **A** showing cluster formation (arrowheads) of recipient DCs (blue) with BrdU^+^ cells (red). Scale bar = 20 μm. **(E)** Three-color immunofluorescence staining of the PALS for XCR1 (green), CD103 (red), and EdU (white) 2 days after DST, showing clusters (arrowheads) of XCR1^+^CD103^+^ (left panels) or XCR1^−^CD103^+^ (right panels) DCs with EdU^+^ proliferating cells (white). Scale bar = 10 μm. **(F)** Proportion of cluster-forming cells in the PALS, showing ~50, ~15, and ~35% were XCR1^+^CD103^+^ DCs, XCR1^−^CD103^+^ DCs, and XCR1^+^CD103^−^ non-DCs, respectively (mean ± SD, *n* = 3 rats each, *P < 0.05).

When activation state of DC subsets was examined 36 h after transfer of CFSE-labeled donor TDLs, CFSE^+^XCR1^+^ DCs showed a significant upregulation of CD40 (Figures 7A,B), although CFSE^−^XCR1^+^ and CFSE^−^XCR1^−^ DCs showed no change (Figure S6). CD103^+^ DCs readily formed clusters with proliferating cells in the PALS 2 days after DST (Figures 7C,D), which was composed mostly of CD4^+^ T cells as reported previously (24). Although we observed selective phagocytosis of donor cells by XCR1^+^ DCs (Figure 3), not only XCR1^+^ DCs (~50%), but also XCR1^−^ DCs (~16%) formed clusters with recipient EdU^+^ proliferating cells in the PALS (Figures 7E,F). Cluster-forming XCR1^+^CD103^−^ non-DCs were also found (Figure 7F) and may be a macrophage lineage.

### Antigen-Labeled Donor T Cells Can Induce Specific Antibody Production

**Figure 8:**
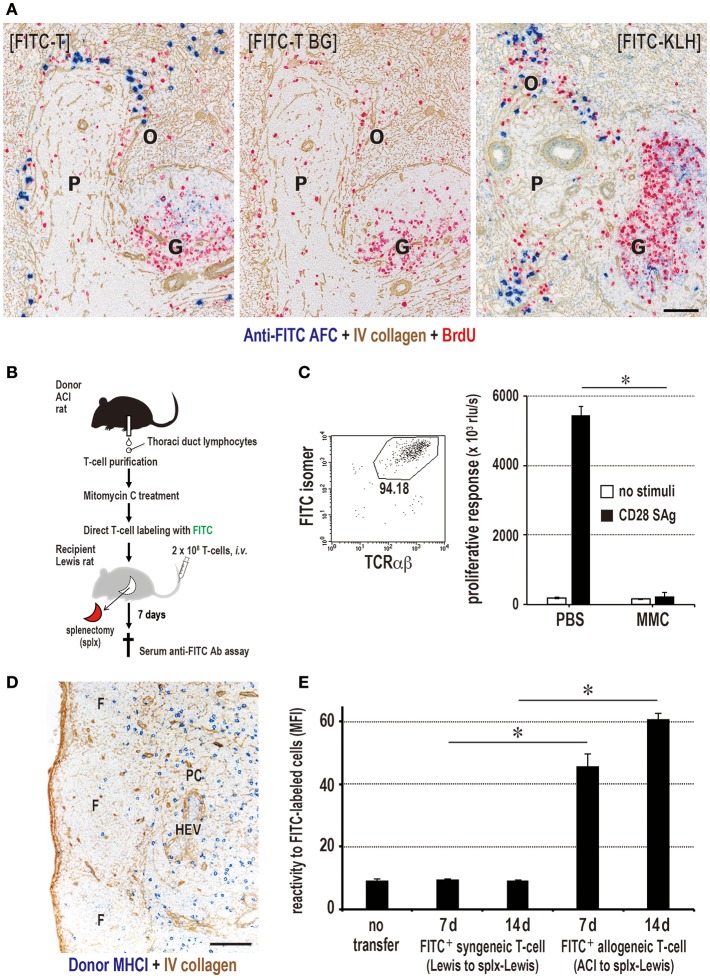
Antigen-labeled donor T cells induce specific antibody production. **(A)** Three-color immunostaining of recipient spleen for anti-FITC antibody (blue), type IV collagen (brown), and BrdU (red). FITC-T cells induced specific anti-FITC antibody forming cell response (blue) in the outer margin of the PALS (O) at 7 days after injection [FITC-T]. When FITC-streptavidin was omitted for background staining, anti-FITC antibody was not detected [FITC-T BG]. Positive control staining for anti-FITC antibody forming cells 5 days after i.v. injection of FITC-labeled KLH [FITC-KLH]. G, germinal center; P, PALS. Scale bar = 100 μm. **(B)** Experimental protocol for inducing antibody production in LNs with FITC-labeled donor T cells. **(C)** Effect of mitomycin C (MMC) on FITC-labeled donor T cell proliferation. Purity of FITC-labeled donor T cells was 94% by FCM. The proliferation of MMC-treated T cells induced by CD28 superagonist (SAg) was abrogated compared to intense proliferation in PBS-treated control. **(D)** Two-color immunostaining for donor MHCI (blue) and type IV collagen (brown) in the LNs 1 d after injection of MMC-treated FITC-labeled donor T cells. They readily migrated to the T cell area of the LNs (paracortex, PC). F, lymphoid follicle; HEV, high endothelial venule. Scale bar = 100 μm. **(E)** MMC-treated FITC-labeled allogeneic, but not syngeneic, T cells induced a low, but significant, antibody response to FITC. **(C,E)** mean ± SD, *n* = 3 rats each, *P < 0.05. MFI, mean fluorescent intensity.

The highly efficient function of T cells in delivering alloantigen to the resident DCs in SLOs and inducing antibody production prompted us to harness donor T cells with antigens as a model of vaccine vectors for prophylactic antibody production. We labeled T cells with either fluorescein isothiocyanate (FITC) or R-phycoerythrin (PE) as a preliminary study. Recipient spleens after injection of labeled allogeneic T cells or soluble FITC-keyhole limpet hemocyanin (KLH) as a control contained specific anti-FITC antibody-forming cells in the outer margin of the PALS, (Figure 8A) and recipient sera contained anti-FITC antibodies (data not shown). When proliferation of FITC-labeled T cells was inhibited by MMC (Figures 8B,C) and i.v. injected into splenectomized rats, they readily migrated to the LNs (Figure 8D). Allogeneic, but not syngeneic, T cells induced anti-FITC antibodies, though their titers were low (Figure 8E).

### Specific Antibody Production Without DST Antibodies in F_1_ Rats That Received Parental Antigen-Labeled T Cells

**Figure 9:**
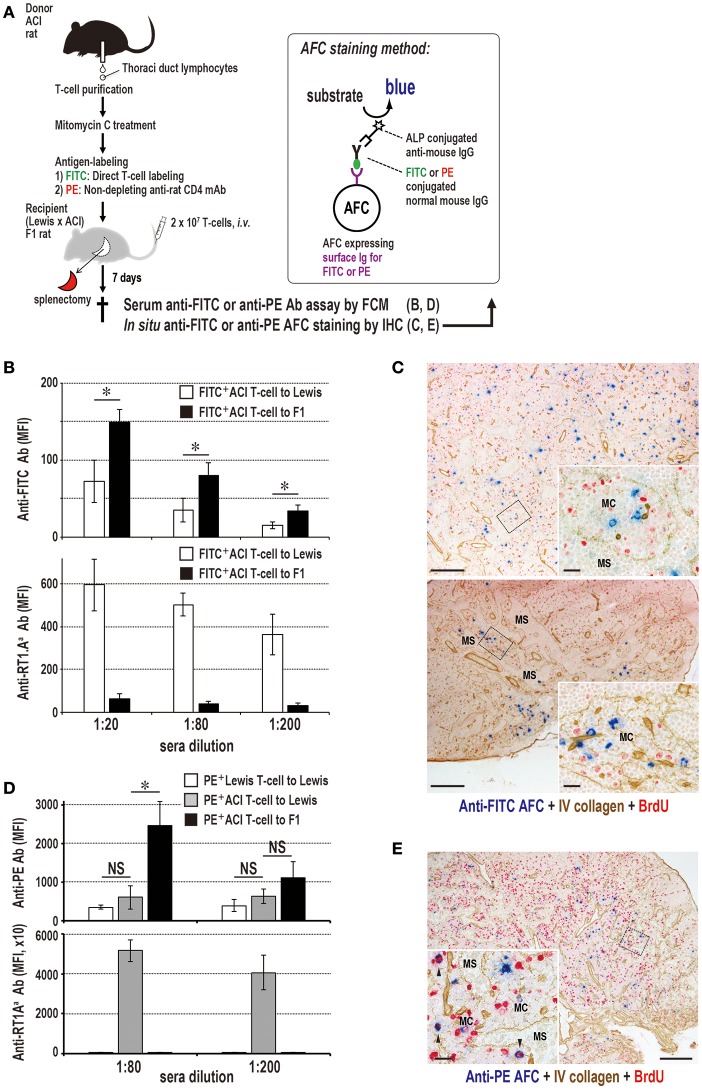
Antibody production to labeled antigens without DST antibodies in F_1_ rats that received parental T cells. **(A)** Experimental protocol for detecting specific antibody production in sera and the LNs. T cells were labeled either with FITC directly or with PE-conjugated ant-rat CD4 mAb. AFC, antibody-forming cells. **(B,D)** Antibody responses in parental ACI (RT1.A^a^B^a^) to (Lewis × ACI)F_1_ hybrid rat (RT1.A^al^B^al^) combination concerning anti-donor MHCI, anti-FITC **(B)**, and anti-PE **(D)** antibodies in recipient sera. Both anti-FITC and anti-PE antibodies of parental to F_1_ rat combination had considerably higher titers than those of the allogeneic control. In contrast, DST (anti-RT1.A^a^) antibodies were negligible, whereas, allogeneic control induced an intense level of DST antibodies (mean ± SD, *n* = 3 rats each, *P < 0.05). MFI, mean fluorescent intensity. **(C,E)** Double immunostaining for anti-FITC (blue, **C**) or anti-PE (blue, **D**) and type IV collagen (brown) in F_1_ rat LNs 7 days after antigen-labeled parental T cell injection. Specific antibody-forming cells (AFCs, blue) against labeled antigen were detected polytopically in the medullary cord of F_1_ rat LNs 7 days after injection. C, cervical (upper panel) and mesenteric (lower panel) LNs; E, mediastinal LNs; MC, medullary cord; MS, medullary sinus. Scale bar = 100 μm **(C,E)** or 20 μm (**C,E** insets).

In order to enhance the antibody response to labeled antigens and to avoid unnecessary alloantibody production, we attempted to inhibit the DST antibody response. We employed the semiallogeneic parental ACI donor and (Lewis × ACI)F_1_ hybrid recipient combination in which recipient T cells (RT1.A^al^) share parental MHCI antigens (RT1.A^a^) and cannot undergo the DST response. In this setting, recipient NK cells could kill parental cells via Ly49c, known as hybrid resistance (35). When MMC-treated and antigen-labeled parental T cells were injected into F_1_ recipients just after splenectomy (Figure 9A), they were readily phagocytosed by XCR1^+^ DCs in the LNs (data not shown) and 7 day sera had considerably higher titers of antibody than the allogeneic recipient group (Figures 9B,D). As expected, DST (anti-donor RT1.A^a^) antibodies were negligible in the semiallogeneic F_1_ recipients, though an intense DST antibody response was induced in the allogeneic recipients (Figures 9B,D). The control group that received equivalent amounts of free PE showed the low level of anti-PE antibodies (Figure S7). Immunohistology of the LNs revealed the polytopical presence of specific anti-FITC or anti-PE antibody-forming cells (Figures 9C,E).

## Discussion

The present study demonstrated several novel findings. First, among donor blood components, T cells were the most proficient immunogens to induce the proliferative responses of recipient CD4^+^ T cells, CD45R^+^ B cells, and Foxp3^+^ regulatory T cells polytopically in the LNs and spleen, followed by serum DST antibody production. Second, donor allogeneic T cells rapidly migrated to the PALS and LNs, followed by a temporary increase in NK cells and the appearance of donor MHCI^+^ fragments, which were selectively phagocytosed by resident XCR1^+^ DCs. Third, inhibition of donor T cell migration or depletion of NK cells significantly suppressed DC phagocytosis and abrogated the DST responses. Fourth, in the T cell areas, EdU^+^ cells, which were mostly proliferating recipient CD4^+^ T cells, originated in the cluster with either recipient XCR1^+^ or XCR1^−^ DCs. Fifth, i.v. administration of antigen-labeled and mitotic inhibitor-treated allogeneic T-cells, but not syngeneic T-cells, to splenectomized recipients induced polytopical specific antibody-forming cell response to labeled antigens in the LNs, as well as the DST response. Finally, split antibody response to labeled antigens without DST antibody response could be induced by employing the semiallogeneic parental donor and F_1_ hybrid recipient combination. The ability of mouse XCR1^+^ DCs to take up apoptotic cells is well documented *in vivo* and *in vitro* (36, 37). We observed phagocytosis of allogeneic lymphocytes *in vivo* by the XCR1^+^DC in mice (38), which will be discussed later.

### Why T-cells Were the Most Efficient and Induced the DST Response Polytopically?

The proficient immunogenicity of donor T cells in the polytopical DST response is most likely due to the ability of blood T cells to transmigrate to the SLOs and contact with resident DCs for immunosurveillance. When normal TDLs of congeneric PVG/c RT7^b^ rats are transferred to PVG/c RT7^a^ rats, they enter the PALS, although not being activated by the DCs (27). Even allogeneic naïve T cells can do so in similar kinetics to that of congeneic T cells (Figure 2) (25–27), thus providing a chance to deliver alloantigen to recipient DCs.

The migration inhibition study with PTX indicate that the presence of donor T cells in the T cell areas is a prerequisite for the DST response. Inability of other blood components that do not enter the T cell areas in a steady state (39) to induce the DST response supports this. Because B cells also enter the T cell area on their way to the lymph follicle, they show a low but significant immunogenicity. Therefore, we consider that the recirculation ability of donor T cells unexpectedly destined them to be the most proficient immunogens for the polytopical DST response.

### Are Killing and Phagocytosis of Donor T-cells Crucial for the DST Response?

The temporary significant increase in recipient NK cells in the PALS and LNs indicates that NK cells are recruited to the T cell areas, probably from the red pulp and the blood, respectively, after the entrance of donor T cells. Active contacts of allogeneic T cells with recipient DCs (27, 35), may lead to activation of DCs via donor T cell receptor (TCR)-recipient MHC and costimulatory molecule interactions (40). The activated DCs may secrete NK-attracting chemokines, such as CXCL10 to recruit NK cells to the LNs as reported (41). However, their increase was under detectable levels by qPCR analysis of the spleen or LNs (Figure S3).

NK cell recruitment was followed by phagocytosis of donor MHCI fragments by XCR1^+^ resident DCs in the T cell areas (Figures 4, 6). The NK-depletion study indicates that phagocytosis of donor cell fragments is dependent on the NK cell recruitment, and the NK strain difference experiment shows that NK activities correlate well with phagocytosis by XCR1^+^ DCs in at least three rat strain combinations. These results suggest that donor T cells were killed by recruited NK cells in the T cell areas prior to phagocytosis. Killing of allogeneic T cells by NK cells is well documented in humans as well as in rats (42).

Syngeneic T cells were also phagocytosed, though to a lesser extent than allogeneic T cells (Figure 4E), suggesting that spontaneous death without NK cell killing may lead to phagocytosis as well. Therefore, we strongly suggest that donor cells are killed mostly by recruited NK cells in at least three rat strain combinations and then phagocytosed by XCR1^+^ resident DCs in the T cell areas. NK-independent killing of donor T cells may also occur.

### Relevance to Our Recent Mice Study

In a mouse two-photon live imaging study of the fate of allogeneic T cells in the SLOs of CD11c-EYFP mice (38), we found a DST antibody response in recipients (unpublished data, Kitazawa), indicating that the DST response in mice is analogous to rats. Donor cell fragmentation and phagocytosis by resident CD11c^+^ DCs in the T cell area of not only the spleen, but also the peripheral LNs, were NK-dependent in at least in some strain combinations.

This study provides several pieces of crucial information. First, allogeneic T cells had more frequent contact with resident DCs with rapid arrest than syngeneic T cells, suggesting that the TCRs of donor T cells have higher affinity for allogeneic MHC on DCs than for autologous MHC. This may promote the clustering of donor T cells and recipient DCs, leading to the activation of DCs and T cells. In other words, live allogeneic T cells may possess some adjuvant effects on the DST response via recipient DC activation. Second, fragmentation of donor T cells was observed extracellularly from 8 h, followed by phagocytosis of donor cell fragments by resident DCs in the T cell areas of the SLOs (38). This demonstrates that donor cell killing and phagocytosis mainly occur in the T cell areas. Thus, the mice data strongly support, at least in part, the proposed mechanism of the DST response in rats.

### Role of Migratory DCs

Alternatively, donor T cells may be phagocytosed by migratory DCs, which in turn migrate to the PALS from the marginal zone, the red pulp, or the blood and induce the DST response. In mouse spleen, these DCs can migrate to the PALS in response to LPS (43) or protein antigens (44). Furthermore, it is recently reported that two mice DC subsets selectively migrate to different areas of the PALS, in response to protein antigens or transgenic erythrocytes (45). Although XCR1^+^ DCs are also found in the rat marginal zone and red pulp, the PTX and NK recruitment experiments in the present study and our recent mouse study (38), demonstrating that donor cell killing and phagocytosis mainly occur in the T cell areas, strongly suggest that XCR1^+^ migratory DCs outside the PALS are not responsible for the DST response.

In regard to the LNs, no study has yet reported that blood mature DCs can perform blood-borne migration to these SLOs via the blood vessel wall (17). Although peripheral antigen-bearing DCs can enter the LNs via the afferent lymph (21), phagocytosis of donor T cells in the periphery would be minor because naïve T cells preferentially migrate to the SLOs (46). Therefore, we consider that XCR1^+^ migratory DCs may be not involved in the DST response in LNs after T cell transfer.

A notable exception is the hepatic LNs, which readily receive blood mature DCs, in rats (18–20) and mice (47), lymphocytes (48), and macromolecules (49) from the liver sinusoids via hepatic lymph (hepatic sinusoid-lymph translocation) (50). This is probably because the sinusoidal endothelial cells are discontinuous and have large fenestrations (51). Thus, the hepatic LNs should be appreciated as strategically unique SLOs that antigen-pulsed DCs in the blood can enter in addition to the spleen.

Taken together, the results indicate that the participation of migratory DCs in phagocytosis of donor T cells is minor. These DCs may be involved in the delayed and less intense DST response in the spleen against blood components other than T and B cells.

### The Indirect Pathway of Allorecognition Operates the DST Response

There are five possibilities as to how the allorecognition occurs in the splenic PALS: the indirect pathway induced by recipient resident DCs (40) in which the allogeneic MHC molecules are presented as peptides in the context of self-MHC; the indirect pathway induced by migratory DCs (45); the direct pathway induced by donor DCs (14, 15); the direct pathway induced by donor T cells via GvH reaction (27, 35); and the semidirect pathway (52).

The indirect pathway induced by migratory DCs and direct pathways induced by donor DCs or T cells were unlikely in the present study. As for the semi-direct pathway, it is operated by recipient DCs that have acquired intact MHCI molecules from donor cells (52), including human activated lymphocytes (53). As donor T cells express MHCI but mostly not MHCII, the recipient DCs could acquire only intact MHCI molecules, which could activate CD8^+^ T cells but not CD4^+^ T cells. Because the CD4^+^ T cell response and antibody-forming cell response against donor MHCI antigens were the major outcomes in this study, the semi-direct pathway is also unlikely in the present study.

**Figure 10:**
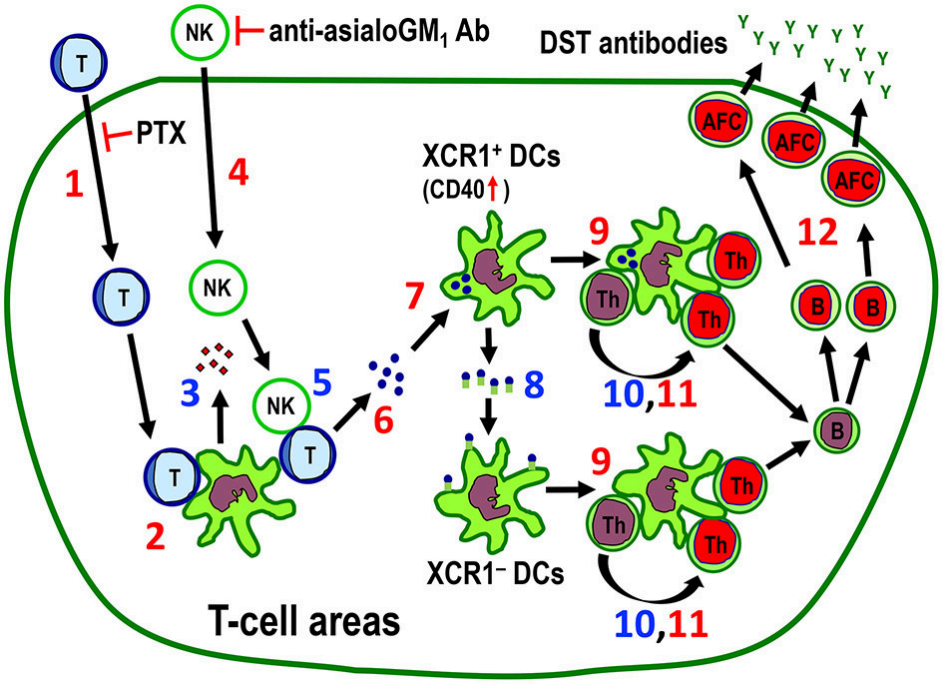
The proposed mechanism for the indirect pathway of allorecognition in the T cell areas of the spleen and LNs. Numbers in red (1, 2, 4, 6, 7, 9, 11, 12) are confirmed findings and those in blue (3, 5, 8, 10) are suggestions. **1**. Migration of injected donor T cells to the T cell areas. Pertussis toxin (PTX) inhibits this process. **2**. Clustering of donor T cells with recipient DCs. **3**. Secretion of NK-recruiting chemokines by recipient DCs. **4**. Recruitment of NK cells to the T cell areas. Anti-asialo GM_1_ antibody (Ab) inhibits this process. **5**. Killing of donor T cells by NK cells. **6**. Fragmentation of T cells in the T cell area. **7**. Phagocytosis of donor T cell fragments by XCR1^+^ DCs, which was associated with upregulation of CD40 in these cells. **8**. Transfer of captured antigenic information by XCR1^+^ DCs to resident XCR1^−^ DCs in the T cell areas. **9**. Cluster formation of both XCR1^+^CD103^+^ and XCR1^−^CD103^+^ DCs in the T cell area. **10**. Presentation of donor MHCI peptides to CD4^+^ T cells. **11**. Proliferative response of CD4^+^ T cells in the cluster (24). **12**. Antibody-forming cell response with production and secretion of donor-specific transfusion (DST) antibodies (24). Blue cells and green cells are donor and recipient cells, respectively. Red nuclei indicate proliferating cells. AFC, antibody-forming cell; Th, CD4^+^ T cell.

If it is the indirect pathway induced by recipient resident DCs, the following sequential steps should be observed: migration of donor cells into the T cell areas; killing or death of donor cells; phagocytosis of donor cells by resident DCs and processing of donor MHCI peptides; binding of resident DCs with recipient T cells to form clusters, within which recipient DCs present donor MHCI peptides in the context of self-MHCII to recipient T cells; which leads to activation and proliferation of T cells within the cluster, resulting in the CD4^+^ T cell-dependent DST antibody response. For the most part, these conditions were observed in the present study (Figure 10). We have already demonstrated the final step (24). For the preceding steps, XCR1^+^ DCs may present donor MHCI antigens after phagocytosis and processing of donor T cell fragments. Upregulation of CD40 in these DCs indicates activation induced probably by the phagocytic event. Surprisingly, however, both XCR1^+^ DCs and XCR1^−^ DCs formed clusters with proliferating CD4^+^ T cells in the PALS (Figure 7). We speculate that, to induce a broad alloimmune response, XCR1^−^ DCs may receive antigenic information from the antigen-containing XCR1^+^ DCs, probably in the form of extracellular vesicles, such as exosomes containing allo-MHCI peptide-recipient MHC complex (54). Although XCR1^+^CD8^+^ DCs in mice are mainly involved in cross presentation, leading to a CD8^+^ cytotoxic T cell response, they can also induce a CD4^+^ T cell response with intense antibody production (55, 56).

Taken together, the results show that the DST antibody response is mainly induced by donor T cells that migrate to the T cell areas via the indirect pathway of allorecognition induced by recipient resident DCs, where migrating donor T cells act solely as alloantigen ferries (Figure 10).

### How B Cells Respond to the Antigen

Although we have not examined where and how the responding B cells gain access to the antigen, there are two possibilities. One is in the PALS, where B cells pass through and may acquire freely floating donor MHCI fragments via antigen-specific receptors. Another is in the B-T boundary (outer margin of the PALS, outer PALS) or periphery of the lymph follicle (outer follicle) (57), where B cells may acquire the antigens. Antigen-specific CD4^+^ T cells activated by antigen-bearing resident DCs in the PALS may encounter the antigen-binding B cells either in the PALS or in the outer PALS, leading to their activation. In this respect, the early accumulation of antigen-binding B cells into the B-T boundary via a new chemokine axis in mice (57) as well as the appearance of BrdU^+^ B-cells in the outer PALS in our recent study (24) suggest that the site for B cell differentiation is the outer PALS.

Concerning B cell response in the spleen and LNs, the major immunoglobulin classes of DST antibodies produced in the spleen and the LNs were not defined. The spleen is considered to be mainly engaged in IgM production (58) and marginal zone B cells are known as sole producers of IgM antibodies to encapsulated bacteria (58). In fact, splenectomized rats showed a considerable decrease of DST antibodies of IgM class, whereas IgG was not affected as previously reported (24). Although these suggest that the major immunoglobulin class produced by antigen-specific B cells in both organs after DST may be different, it may depend upon a type of antigens.

### T-cells Can Be a Vaccine Vector?

The present results imply that antigen-pulsed and mitotic inhibitor-treated allogeneic, but not syngeneic, T cells may be applicable as vaccine vectors without a risk of GvH disease for prophylactic antibody production, especially in asplenic or hyposplenic individuals. Although DST antibodies and/or donor-specific regulatory T cells may be profitable for DST-induced immunosuppression following allotransplantation (24), the active antibody response to conventional antigens is an unexpected benefit. In this respect, if antibody response to labeled antigens were split off from the DST response, as shown in the semiallogeneic parental donor and F_1_ recipient combination (Figure 9), allogeneic T cells may become more convenient vectors that induce only specific antibody response to labeled antigens. The semiallogeneic combination study suggests that donor parental T cells may mimic MHCI-deficient cells with intact TCR molecules, which provides the possibility that MHCI-deficient T cells may be used as ferries of labeled antigens without inducing the DST response. We are now planning to test this working hypothesis by preparing MHCI-deficient allogeneic T cells and label viral antigens.

### Relevant DC Targeting Studies

A few reports have proposed autologous T cells as a vaccine vector for induction of a Th-1 anti-tumor response (59, 60). Although the concept of using recirculating T cells is similar to ours, the clinical outcomes using autologous cells were underwhelming (59). In our study, spontaneous death and phagocytosis of syngeneic T cells by DCs were also observed (Figure 4E), whereas antibodies against pulsed antigens were not detected. To obtain the appropriate response, strong adjuvants would be required (60), which may lead to undesirable side effects.

It has also been reported that, following infusion of ganciclovir-sensitive suicide gene-transduced donor allogeneic T cells to human hematopoietic stem cell transplantation recipients, T cell responses to the gene products are rapidly induced and coincide with the disappearance of transferred cells (61). Although the mechanism of this recipient immune response and the polytopical antibody-forming cell response have not been examined, this response may be relevant to our study.

As an alternative to targeting DCs, allogeneic fibroblasts were used as adjuvant vector cells to furnish viral antigens and alpha galactoceramide, which readily induced invariant NK T-cell-dependent killing and phagocytosis of donor cells by immature DCs in the marginal zone with an efficient antibody response against viral antigens (62). Because adjuvant vector cells are fibroblasts, which may not migrate to the LNs, only a splenic response may occur.

The selective targeting of DC-specific endocytic receptors by linking the antigens to antibodies is most widely studied approach to activate T cells (3). Although few report is available concerning the immune response in the LNs, these antibodies may extravasate at the peripheral organs and enter the LNs via the afferent lymph in the same fashion as other plasma proteins (9). However, because they often require adjuvants for inducing strong and persistent immune responses and avoiding T cell tolerance (3), well-designed DC targeting antibodies coupled with antigens and adjuvants that can reach the LNs would be necessary for the polytopical responses.

Taken together, we consider that no report is yet available except for the present study concerning DC targeting using allogeneic T cells for induction of polytopical antibody responses in the LNs.

## Conclusion

We demonstrate that T cells in donor blood behave as self-migratory alloantigens delivered to the resident XCR1^+^ DCs in the T cell areas of SLOs and induce the indirect pathway of allorecognition, which leads to the polytopical recipient CD4^+^ T cell-dependent response, with efficient alloantibody production without adjuvant. Clinically, transfusion of mitotic inhibitor-treated allogeneic blood T cells pulsed with antigens may be applicable as a vaccine vector for prophylactic antibody production without the risk of GvH disease. This method may be especially helpful for asplenic or hyposplenic individuals (63) to whom non-live vaccines may only be effective in the regional LNs.

## Materials and Methods

### Animals

Inbred ACI rats (RT1.A^a^B^a^) were obtained from the National Bio Resource Project for the Rat in Japan (Kyoto, Japan). Lewis rats (RT1.A^l^B^l^), DA rats (RT1.A^a^B^a^), BN rats (RT1.A^n^B^n^), and PvG/c rats (RT1.A^c^B^c^) were supplied by SLC Japan, Inc. (Shizuoka, Japan). RT1.A and RT1.B are rat MHCI and MHCII antigens, respectively. ACI rats and (Lewis × DA)F_1_ and (Lewis × ACI)F_1_ hybrid rats (RT1.A^al^B^al^) were bred at Dokkyo Medical University Animal Research Center (Mibu, Japan). All rats were reared under specific pathogen-free conditions. Animal handling and care were approved by the Dokkyo Medical University Animal Experiment Committee in accordance with the university's regulations for animal experiments and Japanese law (No. 451). In most experiments, ACI rats donated blood and TDLs and Lewis rats were recipients. BN and PvG/c rats were used as recipients in the strain difference experiment. (Lewis × DA)F_1_ and (Lewis × ACI)F_1_ rats were also used as donors and recipients in semiallogeneic combination experiments, respectively.

### Antibodies and Reagents

Antibodies and probes used for immunohistology, FCM sorting, and immunomagnetic bead sorting are listed in Table S1. Some mAbs were purified from culture supernatants and coupled to FITC, PE, PerCP/Cy5.5 (Innnova Bioscience Ltd, Cambridge, UK), Alexa-Fluor 488, 594, or 647 (Molecular Probes, Eugene, OR) in house.

### Experimental Design

In general, freshly heparinized whole blood was aseptically obtained from the donor abdominal aorta and blood components fractionated and injected i.v. into the tail veins of recipient rats. In some experiments, we used TDLs as a source of donor lymphocytes. By routine thoracic duct cannulation of adult rats (64), up to 10^8^ TDLs could be obtained by overnight collection, including mature peripheral T cells (~80%), B cells (~20%), and non-lymphoid cells (< 2%) with a viability of >95%. At various times from 1 to 14 days after cell transfer, recipient rats received an i.v. injection of 5-bromo-2'-deoxyuridine (BrdU, 2 mg/100 g body wt; Sigma Aldrich Japan, Tokyo) for immunoenzyme staining, or ethynyl deoxyuridine (EdU, 2.5 mg/100 g body wt; Life Technologies Japan, Tokyo) for immunofluorescence staining and FCM of proliferating cells 1 h before sacrifice under isoflurane anesthesia. The SLOs, including the spleen, peripheral LNs (cervical, axillary, and brachial LNs), gut LNs (mesenteric LNs), hepatic LNs, and mediastinal LNs, were excised and fresh-frozen for immunohistological examination (24, 35). Samples of the spleens and LNs were digested by collagenase D (Roche Diagnostics, Indianapolis, IN) and the cells used for FCM. Stained cells were acquired on Attune NxT flow cytometer (Thermo Fischer Scientific Inc.) or FACSCalibur (BD Biosciences) and data were analyzed with FlowJo ver.9.2 (FlowJo LLC, Ashland, OR). Sera were collected and heat inactivated for 30 min at 56°C for detection of antibodies.

We performed five specific experiments. First, by separating blood components, effective components for the DST response were surveyed by FCM (24). Second, the fate of donor cells and a role of recipient phagocytic DCs and NK cells in the spleen were studied *in vivo* using multicolor immunohistological staining or FCM. Third, the mechanism underlying the DST response was examined regarding how the responsible blood components worked and the cluster formation by recipient DCs and CD4^+^ T cells. Fourth, DST responses in other SLOs were examined mainly in splenectomized recipients. Finally, as a model of a vaccine vector for prophylactic antibody production, mitotic inhibitor-treated donor T cells were labeled with antigens and antibody-forming cell responses against them examined mainly in splenectomized recipients.

### Isolation of Blood Components

To clarify the blood components responsible for DST antibody production, donor blood was fractionated into WBCs, T cells, platelets, RBCs, and plasma. The cell dose was adjusted to the equivalent numbers as those contained in 1 ml of whole blood. The volume of plasma injected was 1 ml (See Online Supplemental Material). For WBC and platelet isolation, heparinized blood was centrifuged at 1,600 *g* for 10 min at room temperature and a buffy coat containing WBCs and platelets, a pellet containing mainly RBCs, and the supernatant with cell-free plasma were collected separately. The buffy coat was centrifuged at 1,100 *g* for 20 min at room temperature on a density gradient medium, Lympholyte-Rat (Cederlane Inc., Ontario, Canada), to remove contaminating RBCs. The interface was collected and centrifuged at 110 *g* for 10 min. The pellet contained mostly WBCs and the supernatant was mostly platelets. After repeating the centrifugation, the WBC and platelet fractions were collected. The purity of both fractions was >99% with negligible contamination by RBCs. For the WBCs without T cells, the WBC fraction was treated with a cocktail of anti-CD3 (Biolegend, San Diego, CA) and TCRαβ (Biolegend) and then negatively selected using auto MACS (Miltenyl Biotech, Gladbach, Germany). This depleted T cells to 0.1% of total T cells.

For T cells, small lymphocytes were enriched from the above WBC fractions on discontinuous gradients of OptiPrep (Axis-Shield, Oslo, Norway) made by overlaying 3 ml of 11.5% OptiPrep in HBSS onto 4 ml of 15% OptiPrep solution in a 15-ml conical centrifuge tube. A total of 1 × 10^7^~10^8^ WBCs/6 ml of PBS were overlaid onto gradients and centrifuged at 600 *g* for 20 min at room temperature. The interface between the medium and 11.5% OptiPrep containing large cells, such as DCs and monocytes, was discarded. The interface between 11.5 and 15% OptiPrep containing mainly small lymphocytes was collected. T cells were purified by passing the lymphocytes through the nylon-wool column (Polysciences Inc., PA, US), with a resulting purity ~95% (65). Contaminating cells were mostly B cells. For further isolation of CD4^+^ or CD8^+^ T cells, purified T cells were incubated with FITC-conjugated anti-CD8α or anti-CD4 antibodies and Alexa-Fluor 647-conjugated anti-MHCII antibodies, and then sorted by FACSAria II (BD Bioscience, San Jose CA) as double-negative cells. The purities of sorted CD4 and CD8 T-cells were > 99%. 1.5 × 10^6^ cells of whole T-cells or T-cell subsets were injected i.v. to recipient rats.

For RBCs, the blood pellet was centrifuged repeatedly on Lympholyte-Rat to eliminate the remaining leukocytes. After this, contaminating WBCs were < 10 cells/10^7^ RBCs.

Concerning the dose response to donor T cells and B cells, small lymphocytes from TDLs were enriched on discontinuous gradients of OptiPrep and T cells purified as described above. B cells were purified by negative selection with mAbs to CD4 (Biolegend), CD8β (Thermo Fisher Scientific Inc.), CD11b/c (Biolegend), and CD161a (BD Biosciences) using FACSAria II (BD Biosciences). A total of 10^5^, 3 × 10^5^, and 10^6^ T or B cells isolated from TDLs were i.v. injected into Lewis rats.

### Serum DST Antibody Detection

Serum alloantibody was detected as described previously (24) with a slight modification. In brief, 2.0 × 10^5^ TDLs from donor ACI rats were incubated with DST-treated sera for 30 min at 4°C. After washing twice, the cells were incubated with the cocktail of FITC-conjugated anti-rat IgM and PE conjugated anti-rat IgG Fc for 30 min at 4°C. Then, the cells were further stained with Alexa-Fluor 647-conjugated anti-rat TCRαβ antibody for 30 min at 4°C in order to gate donor T-cells as targets, because donor B-cells express surface Ig, which could cross-react with secondary antibodies. Target cells were analyzed by FCM (BD Biosciences) and the reactivity of alloantibody was estimated as mean fluorescent intensity (MFI).

### Multicolor Immunoenzyme and Immunofluorescence Stainings

Fresh 4-μm-thick cryosections of target organs were multicolor immunostained as described previously (24, 66, 67). Briefly, for three-color immunoenzyme staining, donor MHCI or leukocyte markers (alkaline phosphatase, blue), type IV collagen or recipient MHCII (peroxidase, brown), and BrdU (alkaline phosphatase, red) were detected. For 4-color immunofluorescent staining, donor MHCI (Alexa-Fluor 594, red), leukocyte markers (Alexa-Fluor 647, infrared), type IV collagen (AMCA, blue), and secondary leukocyte markers, MHCI, or EdU (Alexa-Fluor 488, green) were detected. Multichannel images were captured using an Axioskop 2 Plus fluorescence microscope equipped with an AxioCam MRm camera (Zeiss, Oberkochen, Germany). We assigned pseudocolors to each channel to increase the clarity of merged images by maximizing contrast using AxioVision software (Zeiss).

### Phenotype of Phagocytic DCs in the T Cell Area

As phagocytosis of donor MHCI^+^ fragments by recipient putative DCs in the PALS was suggested, we examined the phenotypes of conventional DC subsets in a steady state and after DST treatment by FCM and immunohistology. Rat conventional DCs are reported to be CD103^+^MHCII^+^ (28, 29), with two subsets present in the spleen: CD4^+^SIRP1α^+^CD200^−^ and CD4^−^SIRP1α^−^CD200^+^ (68). Recently, all conventional DCs in the mouse were reported to be universally subdivided into either XCR1C^+^SIRP1α^−^CD8^+^ DCs or XCR1C^−^SIRP1α^+^CD8^−^ DCs, regardless of their activation status (28). Rat DCs were also reported to be subdivided into either XCR1^+^CD4^−^ DCs or XCR1^−^CD4^+^ DCs (69). Accordingly, these markers were examined together with CFSE-labeled donor cells. DCs were enriched from low density fractions of spleen cells by positive selection with CD103 microbeads and auto MACS. The phenotypes of low density fractions containing CFSE^+^ signals were examined by FCM. These cells were further purified by FACSAria II sorting (BD Biosciences), and cytosmears were stained with three-color immunofluorescence for donor MHCI, XCR1, and DAPI. For immunohistological analysis, the spleen cryosections were stained with 4-color immunofluorescence for donor MHCI, CD103, type IV collagen, and XCR1. For FCM and immunohistology, isotype control of the XCR1 mAb (IgG_2b_) was used to confirm the specificity of the mAb.

To examine activation state of recipient DCs after donor cell phagocytosis, CFSE^+^XCR1^+^ DCs were investigated by multicolor FCM for CD25, CD40, CD54 (ICAM-1), CD80, and CD86. CD25 was reported as one of the activation markers in rat and human DCs (15, 70).

### Role of NK Cells in Donor Cell Phagocytosis in the PALS

To examine the role of NK cells in donor cell phagocytosis, we quantified the number of NK cells in the PALS at 0 ~ 12 h after DST. NK cells and PALS areas were immunohistologically defined as asialo GM${}_{1}^{+}$ cells and the white pulp area encircled by the IgM^+^ area (marginal zone and lymph follicle), respectively, on the spleen cross-sections. The whole areas of the cross sections were photographed and reconstructed using image software (Microsoft Image Composite Editor, Microsoft, Redmond, WA) and the numbers of NK cells/mm^2^ in >50 PALS areas counted. For the LNs, whole LN cell suspensions were prepared and the number of CD161a^+^ asialo GM${}_{1}^{+}$ cells examined by FCM.

Next, we depleted NK cells by i.v. injecting anti-asialo GM_1_ polyclonal antibody (35) (2.5 mg in 1 ml PBS per rat, Wako Pure Chemicals, Tokyo, Japan) at −1, 1, 3, and 5 days after DST. In the untreated spleen, CD161a^+^ NK cells were distributed primarily in the red pulp and marginal zone, and a few in the PALS. The antibody treatment resulted in almost complete disappearance of NK cells in the spleen up to 7 days after DST. After NK depletion, the DST effects concerning serum antibody production and DC phagocytosis were examined.

For detection of NK cell-recruiting chemokines, qPCR was performed for the spleen and peripheral LNs at 0 ~12 h after DST following standard methods (71). Briefly, 100 mg of frozen cervical LNs or splenic tissues were dissolved in 1 ml of ISOGEN (Nippon Gene, Tokyo, Japan) and RNA extracted following the manufacturer's instructions. To improve yield, 1.5 μL of ethachinmate (Nippon Gene) was added during isopropanol precipitation. To ensure removal of genomic DNA, samples were processed with DNase I (TakaraBio, Shiga, Japan). After spectrophotometric quantitation, RNA was reverse-transcribed using PrimeScriptTM Reverse Transcriptase (TakaraBio). RNA integrity was confirmed by agarose gel electrophoresis. qPCR was performed using Universal Probes (Roche, Basel, Switzerland) as reported previously (71). Primer sets were designed according to the Universal Probe Library Assay Design Center (https://qpcr.probefinder.com/organism.jsp) (Table S2). Data were calculated using the ddCt method to obtain the mRNA copy number ratio for the genes of interest against beta-actin.

For strain differences in NK activities, PvG/c rats were used as NK-high responder recipients and BN rats as the other strain whose NK activity was not classified (33). ACI TDLs were injected i.v. into PvG/c or BN rats and donor cell phagocytosis and serum DST antibody responses compared to Lewis rats.

We examined CD161a (NK1.1) and asialo GM_1_ expression in splenic DCs, CD8^+^ T cells, and NK cells and the effect of asialo GM_1_ antibody treatment.

### Pretreatment of Donor Cells

To block the chemokine/chemokine receptor axis, we used pertussis toxin (PTX) (72) as few blocking mAbs were available in rats. Briefly, TDLs were incubated with PTX (250 ng/ml, EMD Chemicals Inc., San Diego, CA) for 2 h at 37°C and transfused after washing twice. This temporarily inhibited the migration of syngeneic T cells to the white pulp with a delay of 1–2 days before the effect is lost (72).

For inhibition of mitotic activity, donor T cells were incubated with MMC (20 μg/5 × 10^7^ cells/ml, Sigma-Aldrich) for 30 min at 37°C, and then washed twice just before administration. This condition was determined by a preliminary experiment. T cell proliferation was induced by culturing 1.0 × 10^5^ cells on a CD28 superagonist-bound plate (5 μg/ml) for 3 days at 37°C in a 5% CO_2_ atmosphere (66) and BrdU^+^ cells detected by ELISA. This pretreatment resulted in negligible proliferation *in vitro*, but did not inhibit the migration of T cells to the SLOs, comparable to untreated control (Figures 8B–D).

### Phenotype of DCs That Form Clusters With Recipient T Cells

We previously reported that the recipient CD4^+^ T cell response originates in the recipient DC/T cell cluster in the PALS after DST and suggested that this cluster is site of the indirect pathway of allosensitization (24). In this study, we examined the phenotype of the cluster-forming DCs in the splenic PALS by 4-color immunofluorescence for CD103, XCR1, type IV collagen, and EdU (66). Cluster-forming DCs were defined as CD103^+^ cells with ≥1 EdU^+^ cells in the PALS (24), and the number of CD103^+^XCR1^+^ and CD103^+^XCR1^−^ cells/mm^2^ in the PALS were counted (*n* = 3). CD103^−^XCR1^+^ cells were counted as well, as these cells also formed the cluster.

### Examination of the DST Response in LNs

In the LNs, the donor T cell entrance, NK cell kinetics, phagocytosis of donor MHCI fragments by XCR1^+^ DCs, proliferative response of CD4^+^, CD8^+^, and Foxp3^+^ regulatory T cells and CD45R^+^ B cells, and serum DST antibodies were examined in the same fashion as in the spleen. NK cell kinetics, phagocytosis, and effect of anti-asialo GM_1_ antibody treatment were examined using whole LN cell suspensions.

### Antibody Response to Antigen-Labeled Donor T Cells in the LNs

For antigen-labeling of donor T cells, FITC can be used as a hapten when conjugated with a carrier protein, such as KLH (73) or carrier polysaccharide (32), and can induce anti-FITC antibody-forming cells in a carrier-dependent manner. As skin DCs can be labeled with FITC by skin painting and induce delayed type hypersensitivity in response to secondary challenge (74), we reasoned that FITC-labeled donor T cells may behave as a hapten-carrier antigen and induce anti-FITC antibody production.

After MMC treatment, 10^8^ purified TDL T cells were incubated with 20 μl of 5 mg/ml FITC isomer I (Sigma-Aldrich) in 2 ml RPMI for 30 min at 37°C and washed. A total of 2 × 10^7^ FITC-labeled T cells were injected i.v. into recipients just after splenectomy. FITC-labeled KLH (400 μg/rat) was used as a positive control (73). Specific anti-FITC antibody forming cell staining was performed as described with minor modifications (32, 73). Briefly, cryosections were incubated with either FITC-labeled streptavidin (5 μg/ml, Biolegend) followed by biotinylated alkaline phosphatase (10 μg/ml, Rockland Immunochemicals, Inc., Limerick, PA), or FITC-conjugated isotype mouse IgG (10 μg/ml, Biolegend) followed by alkaline phosphatase-conjugated anti-mouse IgG (Sigma Aldrich) and colored blue. Next, type IV collagen (peroxidase, brown) and BrdU (alkaline phosphatase, red) were immunostained.

As a second antigen, PE-labeled mouse anti-CD4 mAb was used to label the T cells and the anti-PE response (75) examined in the same manner. Donor T cells were incubated with 5 μg of PE-conjugated non-depleting anti-CD4 mAb for 30 min at 4°C and i.v. injected after MMC treatment. As a control, 5 μg of free PE-conjugated mAb was injected. Specific anti-PE antibody forming cell staining was performed using PE-conjugated isotype mouse IgG (Biolegend) instead of FITC-labeled IgG.

For the assay of serum anti-FITC or anti-PE antibody, TDLs from syngeneic Lewis rat were used and pre-incubated with FITC isomer or PE conjugated anti-rat CD47 antibody for 30 min at 4°C. After incubating with sera as above, the cells were reacted with APC-conjugated secondary anti-rat IgM antibody or with Alexa-Fluor conjugated anti-rat IgG antibody (Biolegend). Finally, T-cell was labeled by incubating with PE or Alexa-Fluor 488 conjugated anti-TCRαβ antibody, respectively, in order to gate donor T-cells. Target cells were analyzed by FCM (BD Biosciences) and the reactivity of the specific antibody was estimated as mean fluorescent intensity (MFI).

### Image Analysis and Statistics

Each parameter was measured in a blinded fashion and expressed as the mean ± SD (*n* = 3–4 rats). For estimation of surface areas, image analysis was performed using digital image analysis software (cellSens, Olympus). For FCM, each assay was repeated three times. Statistical analysis was performed using the Student's *t*-test. For quantification of the number of NK cells in the PALS, data was analyzed by Mann-Whitney *U* test.

## Ethics Statement

This study was carried out in accordance with the university's regulations for animal experiments and Japanese law (No. 451). The protocol was approved by the Dokkyo Medical University Animal Experiment Committee.

## Author Contributions

YK and HU designed and performed experiments and wrote the manuscript. YS, TK, and SU performed immunohistological study. HY, KoM, and NT contributed to experiment design and analysis. KeM designed and interpreted experiments and helped write the manuscript.

### Conflict of Interest Statement

HY is employed by TME Therapeutics Inc.

The remaining authors declare that the research was conducted in the absence of any commercial or financial relationships that could be construed as a potential conflict of interest.

## Supplementary Material

Figure S1 Both CD4^+^ and CD8^+^ T cells are equally efficient in induction of serum DST antibody response. After transfer of isolated donor CD4^+^, CD8^+^, or whole T cells **(A)**, DST antibodies were examined on day 7 **(B)**. (mean ± SD, *n* = 3 rats each, ^*^*P* < 0.05). MFI, mean fluorescent intensity.

Figure S2 Conventional DC subsets in the normal rat spleen. **(A)** In 4-color FCM analysis, XCR1^+^SIRP1α^−^ cells and XCR1^−^SIRP1α^+^ cells were detected in the splenic MHCII^+^CD103^+^ DC fraction. XCR1^+^SIRP1α^−^MHCII^+^CD103^+^ DCs were CD4^−^ and CD200^+^, whereas XCR1^−^SIRP1α^+^MHCII^+^CD103^+^ DCs were CD4^+^ and CD200^−^. Note that both DCs were negative for CD8α. **(B)** Note that MHCII^high^ gate (black rectangle) contains XCR1^low~int^ DCs. Isotype control of the XCR1 mAb shows negative staining, confirming the specificity of the mAb. **(C,D)** Three-color immunofluorescence staining of XCR1 (green), CD103 (**C**, red) or CD169 (**D**, red), and type IV collagen (white) in the PALS. **(C)** The arrowheads indicate XCR1^+^CD103^+^ DCs. **(D)** XCR1^+^ cells in the outer margin of the PALS **(C)** are mostly CD169^+^ macrophages (yellow) but those in the PALS (P) are CD169–, mostly DCs (green, arrowheads). Isotype control of the XCR1 mAb shows negative staining. P, splenic PALS. Scale bar = 20 μm **(C)** or 50 μm **(D)**. **(E)** Proportion of two DC subsets in the PALS, which was defined by type IV collagen staining. More than 100 CD103^+^ DCs in the PALS per rat were examined for XCR1 expression (mean ± SD, *n* = 3 rats each).

Figure S3 **(A,B)** Gene expression of NK-recruiting chemokines. mRNA samples isolated from recipient spleens **(A)** or peripheral LNs **(B)** 0~12 h after donor-specific transfusion (DST) were reverse-transcribed and analyzed by qPCR using a Universal Probe Library system. No analyzed gene exhibited a significant difference 4~12 h after DST (mean ± SD, *n* = 3 rats each). **(C)** Three-color FCM analysis of normal splenocytes from Lewis rats for asialo GM_1_, CD161a, and CD103. Most of the asialo GM${}_{1}^{+}$ cells are CD161a^+^ and do not express CD103, indicating that splenic DCs are asialo GM${}_{1}^{-}$. **(D)** 4-color FCM analysis of normal splenocytes from Lewis rats for TCRαβ, CD161a, CD8α, and asialo GM_1_. T-cells (TCRαβ^+^ CD161a^−^ fraction, right upper panel) including CD8^+^ T cells and TCRαβ^+^CD161a^+^ fraction, probably NKT cells (right middle panel), are mostly asialo GM${}_{1}^{-}$. In contrast, asialo GM${}_{1}^{+}$ cells are either CD8α^+^ or CD8α^−^ (right lower panel).

Figure S4 Fate of donor T cells and phagocytosis by XCR1^+^ dendritic cells (DCs) in three different rat strains with different NK activities. **(A)** Experimental protocol for examining donor cell phagocytosis and serum donor specific transfusion (DST) antibody production. MMC, mitomycin C. **(B)** DST antibodies were induced in all strains examined (BN, PvG, and Lewis rats) though at different intensities (*n* = 3 rats each). MFI, mean fluorescent intensity. **(C–F**) Fate of donor cells in BN and PvG rat spleens. Double **(C,D)** or triple **(E,F)** immunostaining for donor MHCI (blue) and type IV collagen (brown), with/without BrdU (red). In PvG rats **(C)**, donor ACI T cells (blue) quickly disappeared by 2 days after transfer. In contrast, in BN rats **(D–F)**, donor T cells persisted at 2 days **(D)** and showed intense proliferation (inset of **E**, arrows) at 3 days **(E)**, indicating a predominance of graft vs. host (GvH) reaction. With MMC pretreatment **(F)**, donor T cells disappeared and the GvH reactivity was inhibited at 2 days. P, PALS. Scale bars = 100 μm **(C–F**) or 20 μm (inset of **E**). **(G,H)** Phagocytosis of donor ACI T cells by XCR1^+^ splenic DCs of PvG **(G)** and BN **(H)** rats. In an ACI to BN combination, donor T cells were pretreated with MMC before transfer. **(I)** Summary of NK activity, donor cell fate, and donor cell phagocytosis in different rat strains.

Figure S5 Graft vs. host (GvH) reaction is not required for the donor-specific transfusion (DST) response. T cells from (Lewis × DA)F_1_ hybrid rats (RT1.A^al^B^al^) were transferred to parental Lewis rats (RT1.A^l^B^l^) in which the GvH reaction does not occur. DST antibody (anti-RT1.A^a^) production was readily observed 7 days after transfer, which was comparable to allogeneic DA (RT1.A^a^B^a^) to Lewis combination (mean ± SD, *n* = 3 rats each). MFI, mean fluorescent intensity; NS, not significant.

Figure S6 Activation state of recipient DCs after donor cell transfer. **(A)** Two major populations of non-phagocytic DCs were gated as MHCII^+^XCR1^+^ cells (X) and MHCII^+^XCR1^−^ cells (Y, SIRP1a^+^DC), respectively. The expressions of CD25, CD40, CD80, CD86, and ICAM-1 in non-phagocytic XCR1^+^DCs **(B)** and SIRP1a^+^DCs **(C)** were compared to those of the control group without cell transfer (mean ± SD, *n* = 4 rats each).

Figure S7 Equivalent amount of free PE (free PE to F_1_) did not induce specific antibodies. **(A)** Experimental protocol for injecting free form PE (*n* = 3 rats). As a positive control, PE-labeled T cells were injected. **(B)** Anti-PE antibody responses in sera of (Lewis × ACI)F_1_ hybrid recipients. Note free PE could induce a low level of antibodies compared to PE-labeled T cells.

Table S1 Antibodies and probes used in this study.

Table S2 qPCR Primers and probes.
