# Supplementary figures and images for: Novel Targeting to XCR1+ Dendritic Cells Using Allogeneic T Cells for Polytopical Antibody Responses in the Lymph Nodes
Source: Front Immunol. 2019 May 29;10:1195. doi: 10.3389/fimmu.2019.01195 (PMC6548820; doi:10.3389/fimmu.2019.01195)

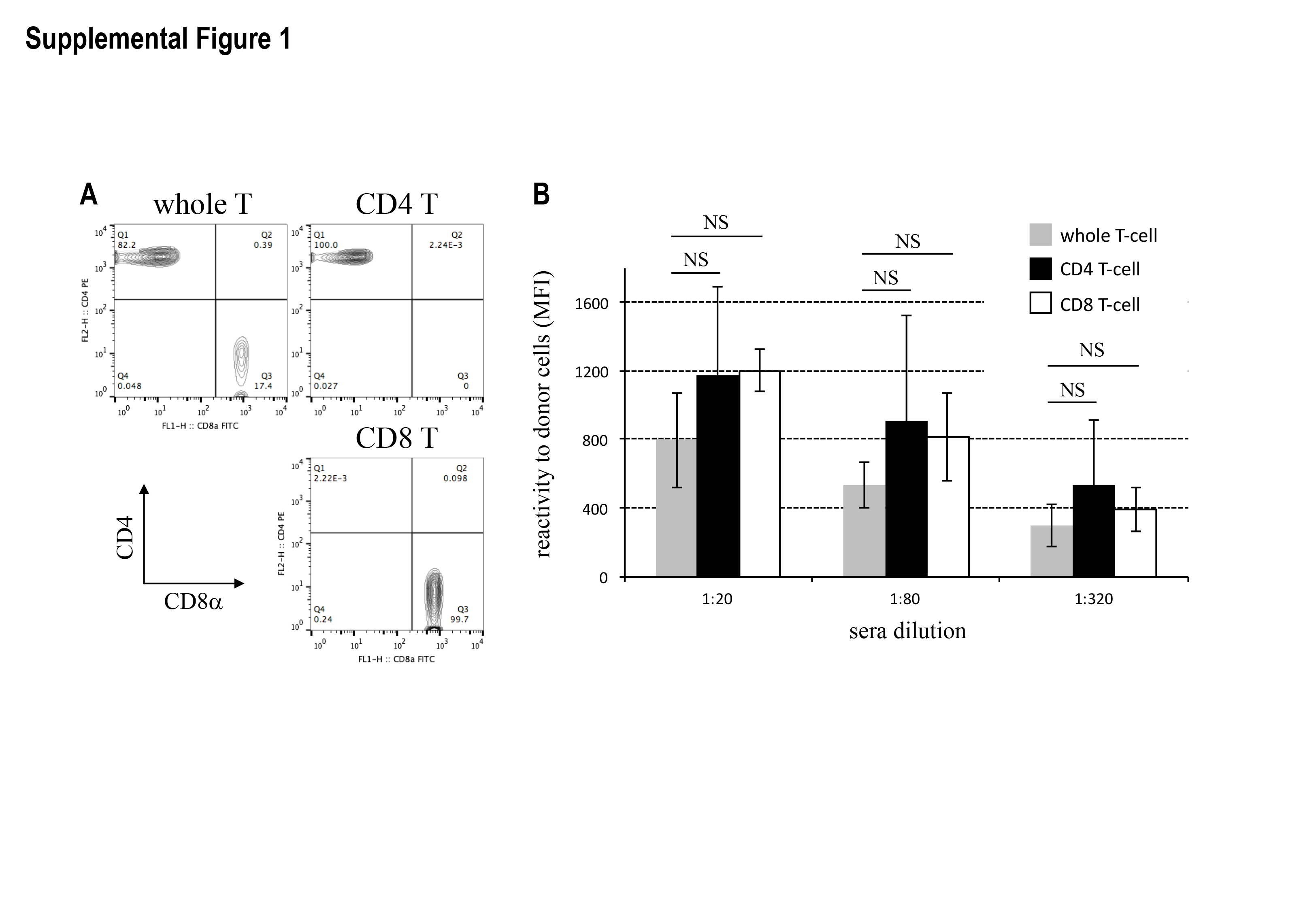

Supplement: Figure S1 — Both CD4+ and CD8+ T cells are equally efficient in induction of serum DST antibody response. After transfer of isolated donor CD4+, CD8+, or whole T cells (A), DST antibodies were examined on day 7 (B). (mean ± SD, n = 3 rats each, *P < 0.05). MFI, mean fluorescent intensity. [file Image_1.TIF]

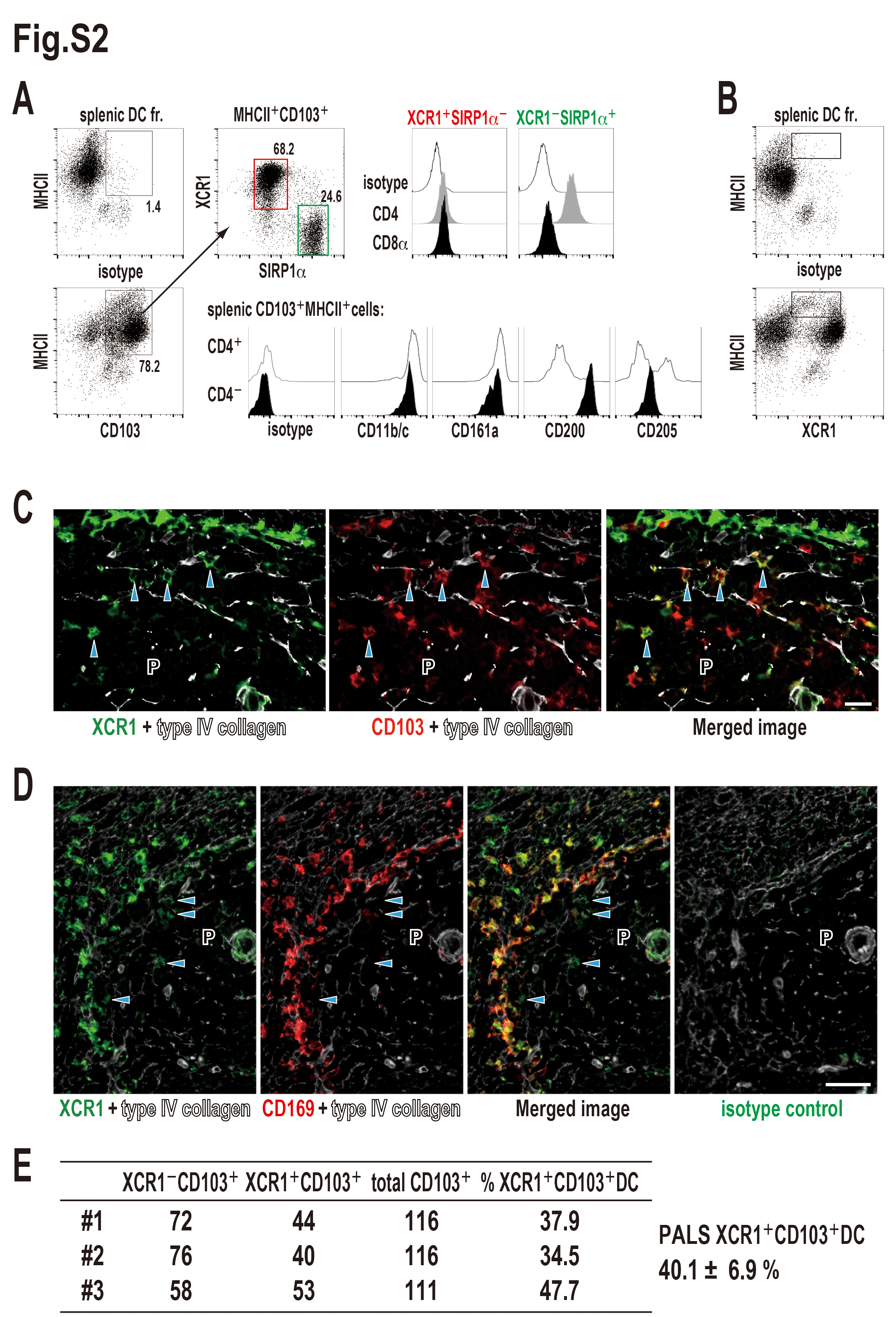

Supplement: Figure S2 — Conventional DC subsets in the normal rat spleen. (A) In 4-color FCM analysis, XCR1+SIRP1α− cells and XCR1−SIRP1α+ cells were detected in the splenic MHCII+CD103+ DC fraction. XCR1+SIRP1α−MHCII+CD103+ DCs were CD4− and CD200+, whereas XCR1−SIRP1α+MHCII+CD103+ DCs were CD4+ and CD200−. Note that both DCs were negative for CD8α. (B) Note that MHCIIhigh gate (black rectangle) contains XCR1low~int DCs. Isotype control of the XCR1 mAb shows negative staining, confirming the specificity of the mAb. (C,D) Three-color immunofluorescence staining of XCR1 (green), CD103 (C, red) or CD169 (D, red), and type IV collagen (white) in the PALS. (C) The arrowheads indicate XCR1+CD103+ DCs. (D) XCR1+ cells in the outer margin of the PALS (C) are mostly CD169+ macrophages (yellow) but those in the PALS (P) are CD169–, mostly DCs (green, arrowheads). Isotype control of the XCR1 mAb shows negative staining. P, splenic PALS. Scale bar = 20 μm (C) or 50 μm (D). (E) Proportion of two DC subsets in the PALS, which was defined by type IV collagen staining. More than 100 CD103+ DCs in the PALS per rat were examined for XCR1 expression (mean ± SD, n = 3 rats each). [file Image_2.TIF]

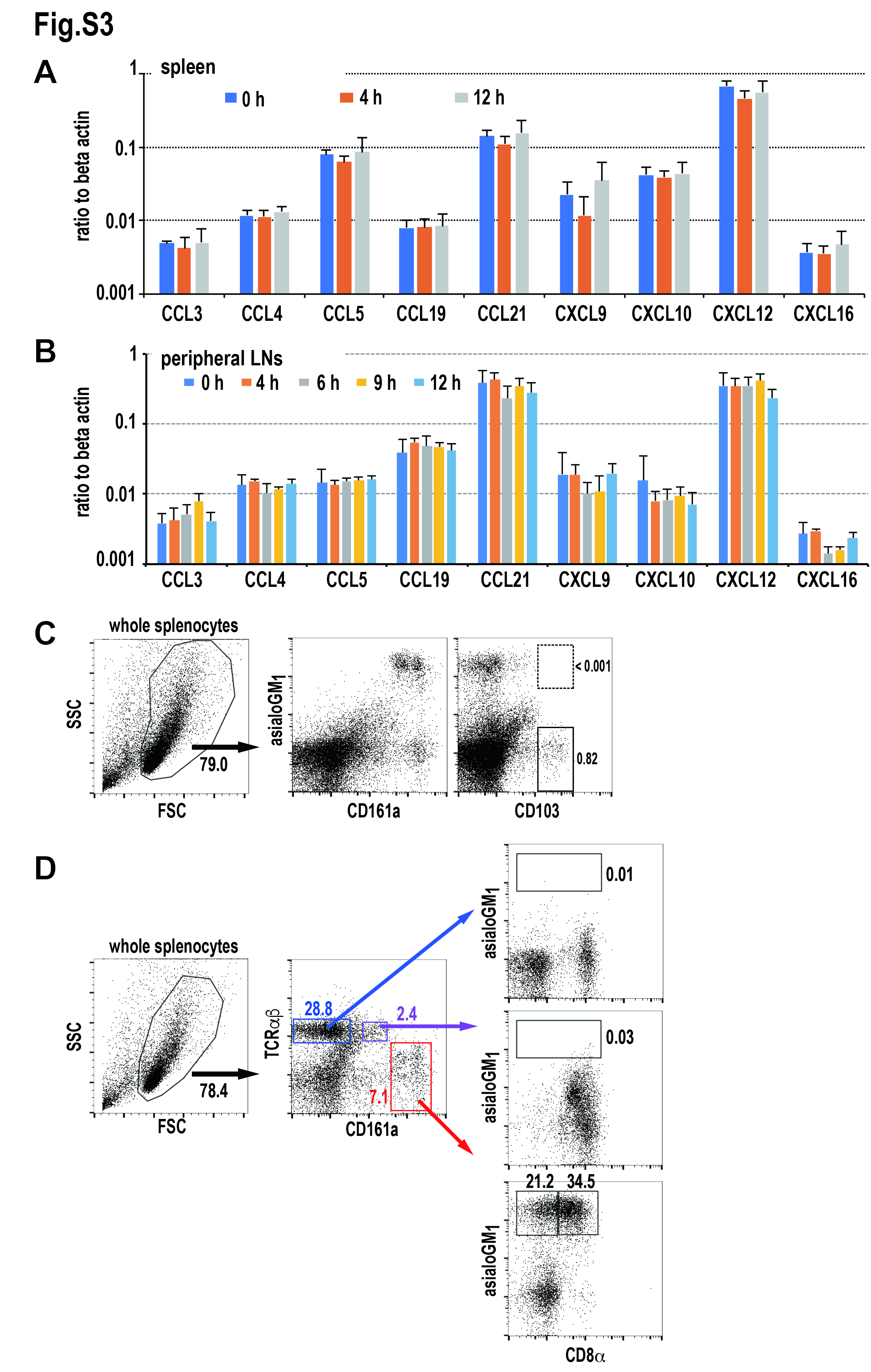

Supplement: Figure S3 — (A,B) Gene expression of NK-recruiting chemokines. mRNA samples isolated from recipient spleens (A) or peripheral LNs (B) 0~12 h after donor-specific transfusion (DST) were reverse-transcribed and analyzed by qPCR using a Universal Probe Library system. No analyzed gene exhibited a significant difference 4~12 h after DST (mean ± SD, n = 3 rats each). (C) Three-color FCM analysis of normal splenocytes from Lewis rats for asialo GM1, CD161a, and CD103. Most of the asialo GM1+ cells are CD161a+ and do not express CD103, indicating that splenic DCs are asialo GM1-. (D) 4-color FCM analysis of normal splenocytes from Lewis rats for TCRαβ, CD161a, CD8α, and asialo GM1. T-cells (TCRαβ+ CD161a− fraction, right upper panel) including CD8+ T cells and TCRαβ+CD161a+ fraction, probably NKT cells (right middle panel), are mostly asialo GM1-. In contrast, asialo GM1+ cells are either CD8α+ or CD8α− (right lower panel). [file Image_3.TIF]

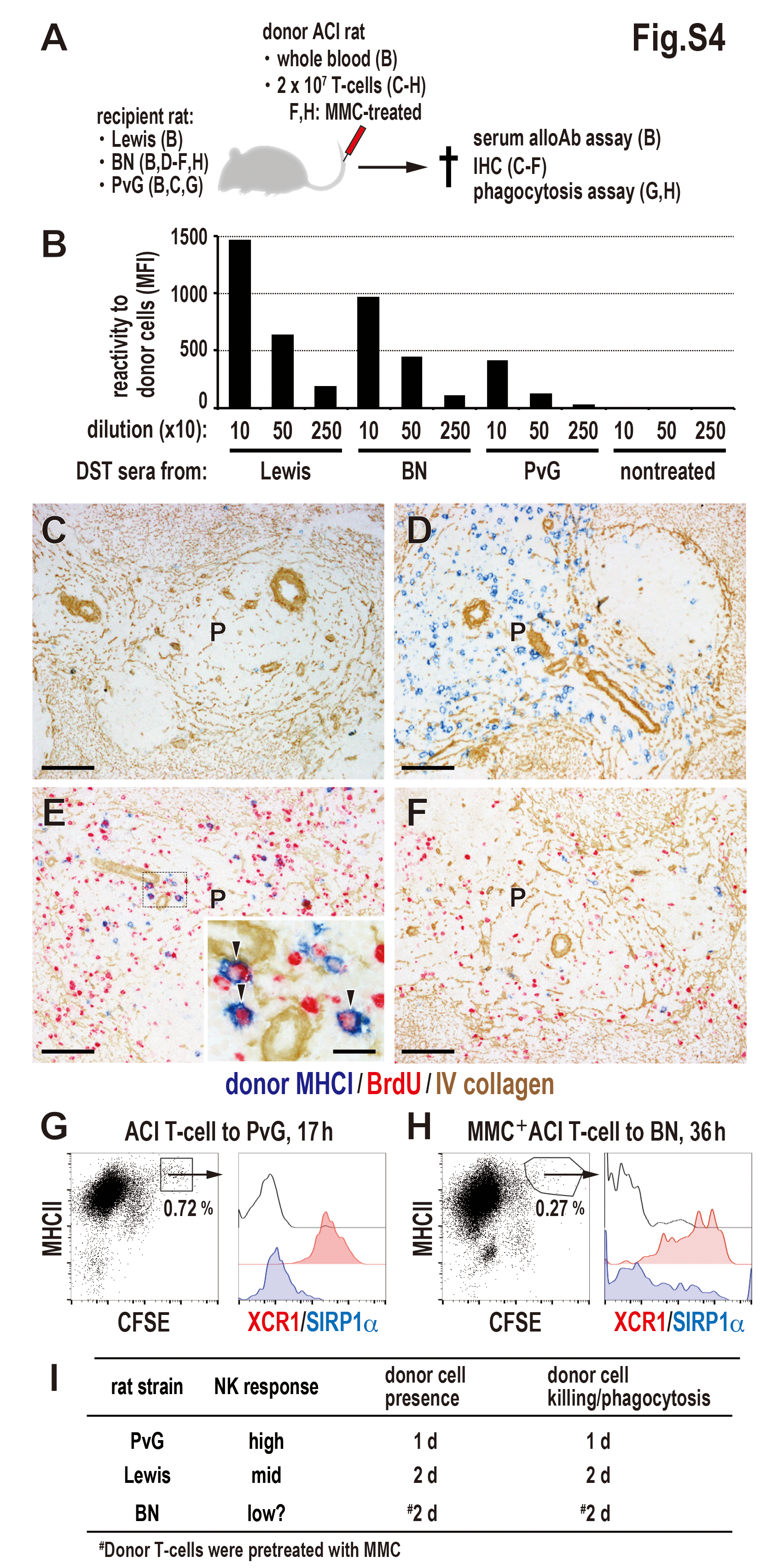

Supplement: Figure S4 — Fate of donor T cells and phagocytosis by XCR1+ dendritic cells (DCs) in three different rat strains with different NK activities. (A) Experimental protocol for examining donor cell phagocytosis and serum donor specific transfusion (DST) antibody production. MMC, mitomycin C. (B) DST antibodies were induced in all strains examined (BN, PvG, and Lewis rats) though at different intensities (n = 3 rats each). MFI, mean fluorescent intensity. (C–F) Fate of donor cells in BN and PvG rat spleens. Double (C,D) or triple (E,F) immunostaining for donor MHCI (blue) and type IV collagen (brown), with/without BrdU (red). In PvG rats (C), donor ACI T cells (blue) quickly disappeared by 2 days after transfer. In contrast, in BN rats (D–F), donor T cells persisted at 2 days (D) and showed intense proliferation (inset of E, arrows) at 3 days (E), indicating a predominance of graft vs. host (GvH) reaction. With MMC pretreatment (F), donor T cells disappeared and the GvH reactivity was inhibited at 2 days. P, PALS. Scale bars = 100 μm (C–F) or 20 μm (inset of E). (G,H) Phagocytosis of donor ACI T cells by XCR1+ splenic DCs of PvG (G) and BN (H) rats. In an ACI to BN combination, donor T cells were pretreated with MMC before transfer. (I) Summary of NK activity, donor cell fate, and donor cell phagocytosis in different rat strains. [file Image_4.TIF]

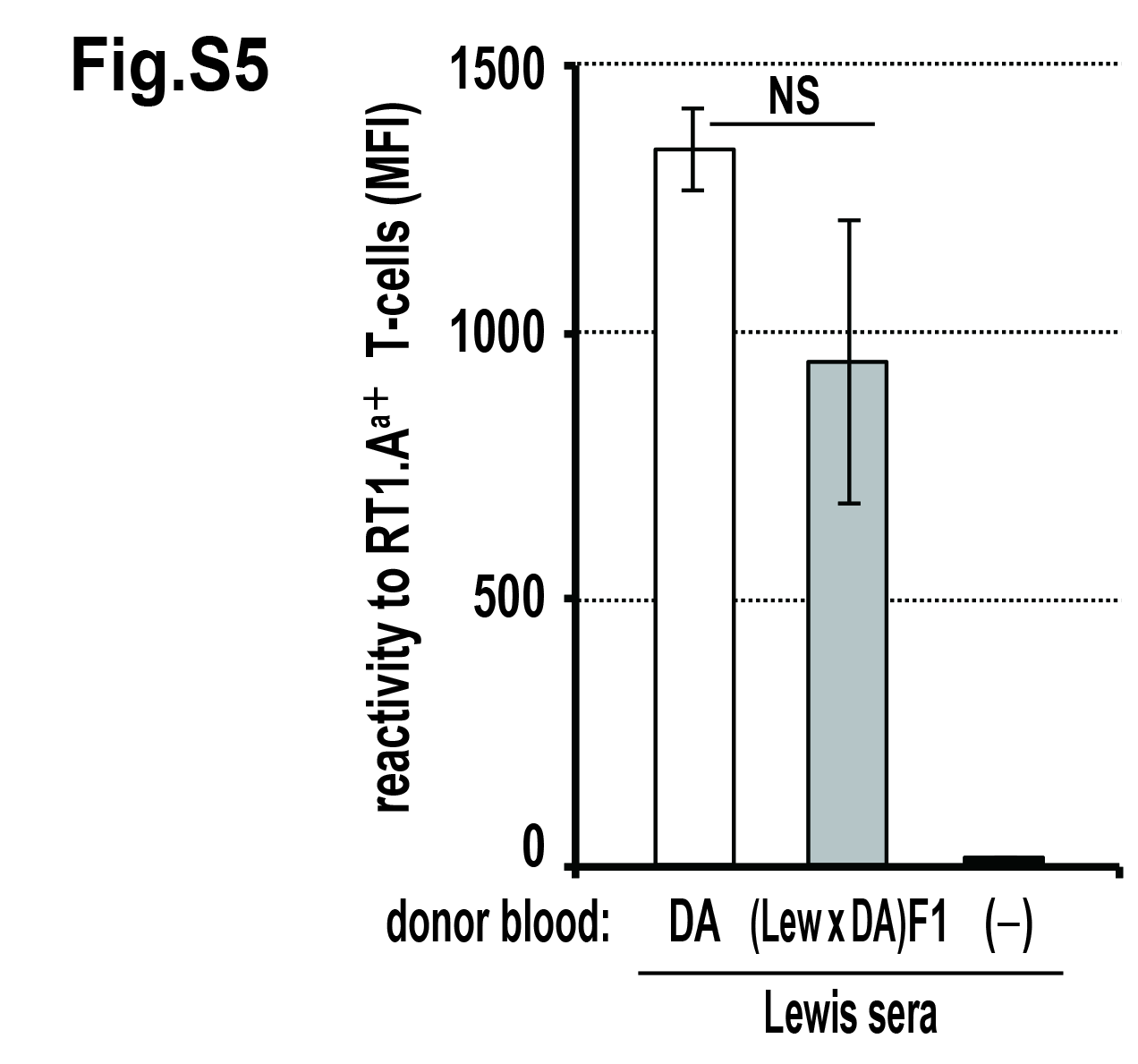

Supplement: Figure S5 — Graft vs. host (GvH) reaction is not required for the donor-specific transfusion (DST) response. T cells from (Lewis × DA)F1 hybrid rats (RT1.AalBal) were transferred to parental Lewis rats (RT1.AlBl) in which the GvH reaction does not occur. DST antibody (anti-RT1.Aa) production was readily observed 7 days after transfer, which was comparable to allogeneic DA (RT1.AaBa) to Lewis combination (mean ± SD, n = 3 rats each). MFI, mean fluorescent intensity; NS, not significant. [file Image_5.TIF]

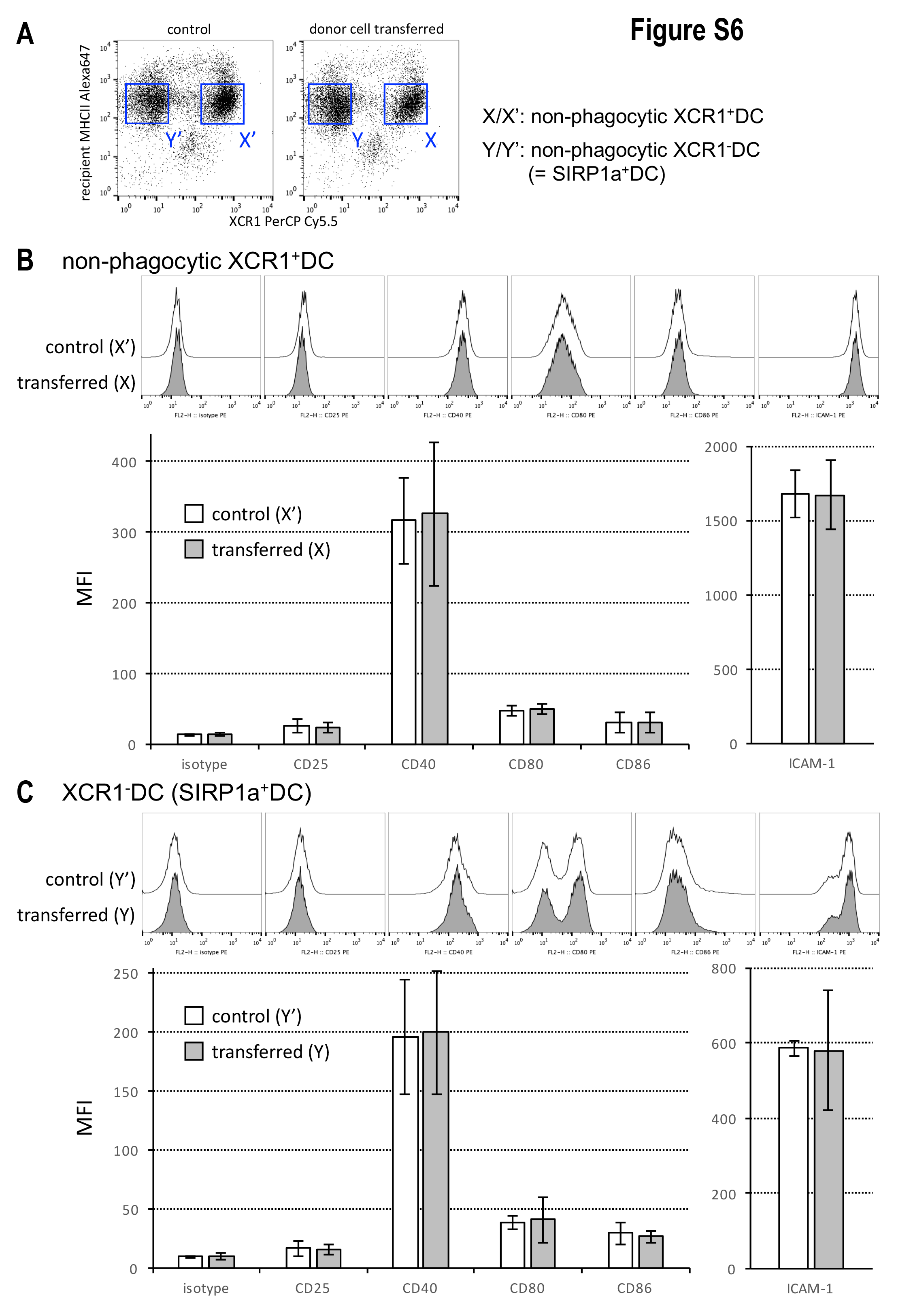

Supplement: Figure S6 — Activation state of recipient DCs after donor cell transfer. (A) Two major populations of non-phagocytic DCs were gated as MHCII+XCR1+ cells (X) and MHCII+XCR1− cells (Y, SIRP1a+DC), respectively. The expressions of CD25, CD40, CD80, CD86, and ICAM-1 in non-phagocytic XCR1+DCs (B) and SIRP1a+DCs (C) were compared to those of the control group without cell transfer (mean ± SD, n = 4 rats each). [file Image_6.TIF]

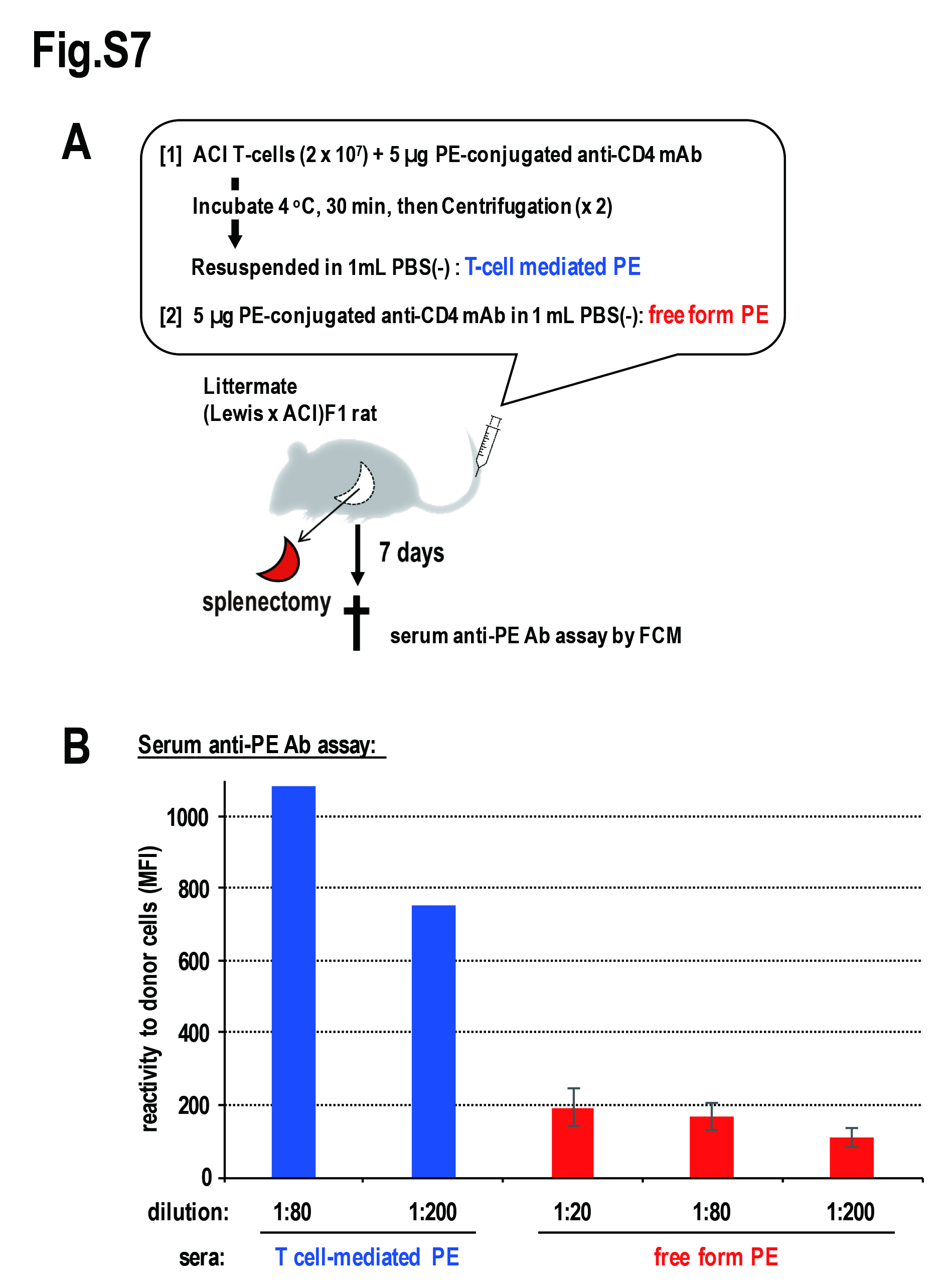

Supplement: Figure S7 — Equivalent amount of free PE (free PE to F1) did not induce specific antibodies. (A) Experimental protocol for injecting free form PE (n = 3 rats). As a positive control, PE-labeled T cells were injected. (B) Anti-PE antibody responses in sera of (Lewis × ACI)F1 hybrid recipients. Note free PE could induce a low level of antibodies compared to PE-labeled T cells. [file Image_7.TIF]
