# Supplementary material for: Novel Targeting to XCR1+ Dendritic Cells Using Allogeneic T Cells for Polytopical Antibody Responses in the Lymph Nodes
Source: Front Immunol. 2019 May 29;10:1195. doi: 10.3389/fimmu.2019.01195 (PMC6548820; doi:10.3389/fimmu.2019.01195)
Supplement: Table S1 — Antibodies and probes used in this study. [file Table_1.DOC]

Table S1. Antibodies and probes used in this study

| 1st Ab and Probes |  | | |  | |
| --- | --- | --- | --- | --- | --- |
| antigen | | clone / probe | source | | conjugate (nil; purified) |
| asialoGM1 | | Rabbit polyclonal | Biolegend, Wako | | Alexa Fluor 647 |
| BrdU | | BU1/75 | AbD Serotec | |  |
| CD4, for flow cytometry | | W3/25 | Biolegend | | FITC, R-PE |
| CD4, for immunohistochemistry | | OX-38 | AbD Serotec | |  |
| CD8α | | OX-8 | Biolegend | | FITC |
| CD8β | | 341 | Biolegend | | FITC |
| CD11b/c | | OX-42 | Biolegend, ECACC*^*^* | | R-PE |
| CD25 | | OX-39 | Biolegend | | R-PE |
| CD40 | | 01 | Sino Biological | | R-PE |
| CD45R (B220) | | HIS24 | Biolegend | | FITC |
| CD47 | | OX-101 | Biolegend | | R-PE |
| CD54 (ICAM-1) | | 1A29 | Biolegend | | R-PE |
| CD80 | | 3H5 | Biolegend | | R-PE |
| CD86 | | 24F | Biolegend | | R-PE |
| CD103 (αE2 integrin) | | OX-62 | Biolegend, ECACC*^*^* | | FITC, Alexa Fluor 647 |
| CD161a (NK1.1) | | 3.2.3 | Biolegend | | R-PE |
| CD172a (SIRP1α) | | OX-41  ED9 | Biolegend  ECACC*^*^* | | R-PE  biotin^#^ |
| CD200 | | OX-2 | Biolegend | | R-PE |
| CD205 (DEC-205) | | HD-83 | Biolegend | | R-PE |
| Foxp3 | | FJK-16s | eBioscience | | Alexa Fluor 647 |
| IgM | | MARM-4 | AbD Serotec | |  |
| normal mouse IgG, for anti-FITC or  anti-PE AFC staining | | MOPC-21 | Biolegend | | FITC, R-PE |
| RT1.A^a^ (polymorphic donor MHCI) | | MN_4-91-6_ | ECACC*^*^* | |  |
| RT1.A^a/c^ (polymorphic donor MHCII) | | OX-76 | ECACC*^*^* | | Alexa Fluor 647^#^ |
| RT1.B^l^ (polymorphic recipient MHCII) | | OX-3 | ECACC*^*^* | | Alexa Fluor 488^#^, 647^#^ |
| RT1.B (monomorphic recipient MHCII) | | 14-4-4s | ATCC^$^ | | Alexa Fluor 647^#^ |
| T-cell receptor αβ | | R73 | Biolegend, ECACC*^*^* | | FITC, PerCP-Cy5.5^#^,  Alexa Fluor 647^#^ |
| XCR1 | | ZET | Biolegend | | R-PE, PerCP-Cy5.5, biotin |
| 5-ethynyl-2'-deoxyuridine (EdU) | | Click-iT® Azide | Life Technologies | | Alexa Fluor 488, 594 |
| type IV collagen | | Rabbit polyclonal | LSL | |  |

| 2nd Abs and Probes | |  | |  |
| --- | --- | --- | --- | --- |
| product | source | | conjugate | |
| biotin | Rockland | | alkaline phosphatase | |
| donkey IgG to rabbit IgG | Jackson ImmunoResearch | | alkaline phosphatase, ^**^AMCA | |
| donkey IgG to rat IgG | Jackson ImmunoResearch | | alkaline phosphatase | |
| goat IgG to mouse IgG | Sigma/ Life Technologies | | alkaline phosphatase, peroxidase/Alexa Fluor 647 | |
| goat IgG to rat IgG | Biolegend | | Alexa Fluor 647 | |
| goat F(ab’)_2_ to rabbit IgG | MP Bioscience | | peroxidase | |
| goat F(ab’)_2_ to rat IgG, Fc | Jackson ImmunoResearch | | R-PE | |
| goat F(ab’)_2_ to rat IgM | Jackson ImmunoResearch | | FITC, allophycocyanin | |
| streptavidin | Biolegend/ Life Technologies | | FITC/ Alexa Fluor 594 | |

^*^The European Collection of Authenticated Cell Cultures, ^$^American Type Culture Collection, ^#^self conjugation

^**^7-amino-4-methylcoumarin-3-acetic acid
