# Supplementary material for: Novel Targeting to XCR1+ Dendritic Cells Using Allogeneic T Cells for Polytopical Antibody Responses in the Lymph Nodes
Source: Front Immunol. 2019 May 29;10:1195. doi: 10.3389/fimmu.2019.01195 (PMC6548820; doi:10.3389/fimmu.2019.01195)
Supplement: Table S2 — qPCR Primers and probes. [file Table_2.DOC]

Table S2. qPCR Primers and probes

| Gene | Primers | | Universal Probe |
| --- | --- | --- | --- |
| *Actb* | Forward | cccgcgagtacaaccttct | #17 |
|  | Reverse | cgtcatccatggcgaact |  |
|  |  |  |  |
| *Cxcl9* | Forward | gccctaactgcaacaaaactg | #115 |
|  | Reverse | tcagcttcttcacccttgct |  |
|  |  |  |  |
| *Cxcl10* | Forward | atgaacccaagtgctgctgt | #13 |
|  | Reverse | gtctcagcgtctgttcatgg |  |
|  |  |  |  |
| *Cxcl16* | Forward | gaactagtgaactgctttgagcac | #113 |
|  | Reverse | gcacatgtttttggtggtga |  |
|  |  |  |  |
| *Cxcl12* | Forward | ccctgccgattctttgag | #21 |
|  | Reverse | tgttgcttttcagccttgc |  |
|  |  |  |  |
| *Ccl3* | Forward | gcgctctggaacgaagtct | #40, |
|  | Reverse | gaatttgccgtccataggag |  |
|  |  |  |  |
| *Ccl4* | Forward | ctctgcgtgtctgccttct | #63 |
|  | Reverse | tgggagggtcagagcctatt |  |
|  |  |  |  |
| *Ccl5* | Forward | ctcaccgtcatcctcgttg | #16 |
|  | Reverse | gagtggtgtccgagccata |  |
|  |  |  |  |
| *Ccl19* | Forward | cccgtgtgaccccactact | #40 |
|  | Reverse | gtcttccgcatcgttagcac |  |
|  |  |  |  |
| *Ccl21* | Forward | ccatcccagcaatcctgtt | #111 |
|  | Reverse | ggcttcctcagggtttgc |  |
